# A Comprehensive Analysis of the Colonic Flora Diversity, Short Chain Fatty Acid Metabolism, Transcripts, and Biochemical Indexes in Heat-Stressed Pigs

**DOI:** 10.3389/fimmu.2021.717723

**Published:** 2021-10-21

**Authors:** Canying Hu, Xueting Niu, Shengwei Chen, Jiaying Wen, Minglong Bao, Sahar Ghulam Mohyuddin, Yanhong Yong, Xiaoxi Liu, Lianyun Wu, Zhichao Yu, Xinbin Ma, Xianghong Ju

**Affiliations:** ^1^ Department of Animal Science, College of Coastal Agricultural Sciences, Guangdong Ocean University, Zhanjiang, China; ^2^ Shenzhen Institute of Guangdong Ocean University, Shenzhen, China; ^3^ Department of Veterinary Medicine, College of Coastal Agricultural Sciences, Guangdong Ocean University, Zhanjiang, China

**Keywords:** heat stress, inflammatory bowel disease, multi-omics, microbiome, short chain fatty acids

## Abstract

Heat stressed pigs show typical characteristics of inflammatory bowel disease (IBD). However, little is known about the pathogenesis of heat stress (HS)-induced IBD in pigs. In this study, we determined the effects of HS on colon morphology, intestinal microbiota diversity, transcriptome genes (transcripts), and short chain fatty acids (SCFAs) metabolism in pigs. In addition, the correlation among these parameters was analyzed by weighted gene co-expression network analysis. Results showed that the liver and kidney functions related to blood biochemical indexes were partially changed in pigs under HS. Furthermore, the levels of diamine oxidase and D-lactic acid were significantly increased, whereas the levels of secretory immunoglobulin A were decreased. The integrity of colonic tissue was damaged under HS, as bleeding, lymphatic infiltration, and villi injury were observed. The concentrations of SCFAs in the colon, such as acetic acid and butyric acid, were decreased significantly. In addition, the composition of colon microbiota, such as decrease in *Lactobacillus johnsonii*, *Lactobacillus reuteri* and increase in *Clostridium sensu stricto 1* of day 7 and 14 while under HS. These changes were associated with changes in the concentration of SCFAs and biochemical indexes above mentioned. Differentially expressed genes were enriched in the nucleotide-binding oligomerization domain-like receptor signaling pathway, Th17 cell differentiation, and IBD pathway, which were also associated with the changes in SCFAs. Thus, the structure, diversity of intestinal microorganisms, and changes in the levels of SCFAs in colon of heat stressed pigs changed significantly, contributing to the activation of immune response and inflammatory signal pathways and causing abnormal physiological and biochemical indexes and intestinal mucosal damage. These results highlight the interconnections between intestinal microbiota, SCFAs, and immune response and their role in the pathogenesis of stress induced IBD therapy.

## Introduction

Heat stress (HS) can cause immunosuppression, intestinal barrier damage, and inflammation in animals that not only affect the digestion and absorption of nutrients and reduce feed reward (1, 2), but also increase the sensitivity of animals to a variety of infectious diseases (3). The heat-neutral range, also called the thermal comfort zone, that the appropriate ambient temperature range when basic heat and heat dissipation of body are balanced, metabolic and physiological functions of animals are at their best, of finishing pigs is 15~22°C (4, 5). In pigs, HS is characterized by intestinal ulcers, mucosal barrier imbalance, and inflammatory cell infiltration (6, 7), which are the typical characteristics of inflammatory bowel disease (IBD). IBD is an inflammatory reaction caused by an imbalance between the immune system and intestinal microecosystem, induced by environmental factors on the basis of susceptible genes (8).

The systemic manifestations of HS are marked by changes in the blood, liver, kidney, and gastrointestinal tract (9–12). Cui et al. found that HS can induce oxidative stress reaction, immune response, and apoptosis of liver cells in pigs, resulting in the damage of liver function (13, 14). Furthermore, oxidative stress caused by HS has been shown to damage mitochondria, cell membrane and protein, and consequently affect the liver lipid metabolism and animal production performance (9). In addition, HS affects the glomerular filtration and urine concentration function of the kidneys by inducing changes in the levels of body electrolytes, especially the balance of sodium, potassium, calcium, and chloride ions, and creatinine metabolism (15, 16).

Several microorganisms, including bacteria, fungi, and viruses inhabit the animal intestines. The ecological community formed by these microorganisms is called intestinal microbiota, which plays an important role in digestion, metabolism, and immune regulation (17–19). When the intestinal flora is imbalanced, the intestinal epithelial cell barrier can be destroyed by the bacterial protease and various toxins produced by the intestinal microbiota (20). Leslie et al. reported that HS led to changes in the intestinal microflora structure and caused diarrhea in pigs (21), but the causal relationship between them was not completely revealed.

Short chain fatty acids (SCFAs) are metabolites of the intestinal microbiota, which are not only the energy source of the host, but also play an important role in regulating mucosal immunity by enhancing mucus secretion and promoting the development of regulatory T cells (22, 23). It has been reported that SCFAs are involved in the maintenance of intestinal function, glucose homeostasis, and appetite and regulation of energy metabolism, inflammation, and immunity and observed in cases of tumors and colon cancer (24–26). At the same time, SCFAs are also related to the host’s gut microbial composition (27, 28). Studies have shown that intestinal microorganisms and their metabolites are involved in the regulation of stress induced IBD (29, 30). Till date, the correlation between intestinal microbiota, SCFAs, and immune response is not fully clear and their role in the pathogenesis of stress induced IBD is unknown.

Here we hypothesize that colonic microbiota and their metabolites may have a potential regulatory relationship with intestinal inflammation under HS. Therefore, in this study, we determined the morphological changes in colon, structure of intestinal microbiota, changes in transcriptome genes (transcripts) and SCFAs metabolism in pigs under heat-neutral range temperature and HS. In addition, the correlation among them was analyzed by weighted gene co-expression network analysis (WGCNA).

## Materials and Methods

### Animals and Management

Total 48 pigs (Luchuan sows × Duroc boars; males), 2-month-old (16 ± 1 kg) were housed in two animal rooms at the Animal Hospital of Guangdong Ocean University, Zhanjiang, China. Pigs with similar body weights were divided into five groups as follows: five pigs each in the day 1, 7, 14 and 21of control groups and HS treatment groups. There were six replicates (one for each sampling date), with three pigs per group. The animals were maintained for 2 weeks at 20 ± 2°C, 75–85% relative humidity, and 12 h/12 h light/dark photoperiod to acclimatize them to the environment. 

**Table:** Groups for experimental animals

Days of treament	Group name	
	Control	HS
1	6	6
7	6	6
14	6	6
21	6	6

Throughout the study, the pigs in each group were served a complete feed formula (Charoen Pokphand Group, China) in the morning, afternoon, and evening in 0.4 kg/serving with approximately 6-h intervals between feedings. Drinking water was freely available. To minimize acute HS, the animal facility was gradually warmed (2° C/d) over a 7-day period. The adaptive feeding continued for 14 days (including 7 days of gradually warming). The HS trial lasted 21 days. The control pigs were exposed to 22 ± 3°C, and the HS-pigs to 34 ± 1°C, at 75–85% relative humidity and 12 h/12 h photoperiod.

The experimental protocols for management and care of pigs were approved by the Animal Care and Use Committee of Guangdong Ocean University, Zhanjiang, China (Permit No. 206-1108).

### Sample Collection

About 5-10 mL of the peripheral blood was collected from each pig through the anterior vena cava. After natural tilt coagulation for 30 min, the blood was centrifuged (3000 ×g) at 4°C for 15 min. Then, the serum was separated and sub packed and stored at -80°C. Subsequently, the pigs were sacrificed by electric shock fainting and bloodletting, and each segment of the colonic tissue was separated by abdominal operation. The sampling position was as consistent as possible. After the separation of adipose and mesentery of colonic tissue, the colonic tissues of each segment were cut and 10 g of the colon content was collected and stored at -80°C. Next, 1-cm segments of the colon were cut and soaked in 10% formaldehyde solution for fixation. The other tissues were cleaned with sterile phosphate-buffered saline (PBS), cut into small pieces, packed in labeled sample bags, and quickly frozen in liquid nitrogen. After collecting, the tissue samples and intestinal contents were transferred to -80°C from liquid nitrogen for cryopreservation.

During the multi-omics test, 3 samples of colonic contents were randomly taken from each group for microbiome experiments sequencing and analysis firstly. Then take the day 1 of heat-neutral range temperature treatment as the control group, analyzed the difference of the biochemical indexes of peripheral blood serum, SCFAs metabolism of colonic contents, and transcriptome of colonic tissues (n = 3) in day 1, 7, 14 and 21 of HS treatment. Finally, analyze the correlation between different indicators of the same pig from each experimental group.

### Determination of Serum Biochemical Indexes

After thawing and centrifugation (3000 ×g at 4°C for 15 min), 200 μL of serum was added to the biochemical analysis systems (Tianjin Mnc Technologies, China), and serum glucose, serum cholesterol, liver function (including albumin, globulin, alanine aminotransferase, and alkaline phosphatase), the renal function (including sodium ion, calcium ion, calcium sodium ratio, chloride, creatinine), creatine kinase, total carbon dioxide, total protein, lactate, inorganic phosphorus, and amylase were determined and analyzed according to the instruction manual (Tianjin Mnc Technologies, China).

### Enzyme-Linked Immunosorbent Assay

Cultural supernatant was stored at -80°C prior to analysis. Serum biochemical markers were measured using ELISA kits for diamine oxidase (MeiMianBio, Wuhan, China; Cat # MM043801), D-lactic acid (MeimianBio; Cat # MM3373201), and secretory immunoglobulin A (sIgA MeimianBio; Cat # MM3623401), according to the manufacturer’s protocol. The intact supernatant was used in the following analysis. The plates were read using a microplate reader (BioTek Instruments Inc., Winooski, VT, USA) at a wavelength of 450 nm. A standard curve for each of the cytokines was used to estimate the concentration. SPSS 19.0 (SPSS, Chicago, Illinois) was used for statistical analysis. The differences among the groups were analyzed by one-way ANOVA, student-T test was used for multiple comparisons. The difference was considered statistically significant for *P < 0.05*.

### Morphological Observations

For histopathological examination, the colonic tissue was fixed in buffered formalin (10% v/v) and stained with hematoxylin and eosin. Image-Pro Plus v. 6.0 (Media Cybernetics Inc., Silver Spring, MD, USA) was used to measure villus height, crypt depth, and width (1). Hydrated colonic tissue sections were treated with amylase at 37°C for 1 h, rinsed under running water for 10 min, and stained with periodic acid solution at room temperature (25 ± 2°C) for 7 min, according to the instructions for the Glycogen D-PAS Staining Kit (Leagene Biotechnology, Beijing, China). The tissue sections were rinsed with tap water, immersed in Schiff’s reagent in the dark for 15 min, and rinsed with tap water for 10 min to remove the stain. The sections were dehydrated with an alcohol concentration gradient (75%, 85%, 95%, and 100%), cleared of alcohol with xylene, and sealed with neutral gum. Image-Pro Plus v. 6.0 (Media Cybernetics Inc.) was used to evaluate the goblet cells per unit area in the colonic mucosa (31).

### Immunofluorescence

Paraffin sections were baked at 55°C and dewaxed thoroughly in xylene, then soaked in gradient alcohol and washed with PBS. The repaired tissues were treated with sodium citrate repair solution at 92°C for 10 min and blocked with 10% fetal bovine serum for 30 min. IgA (1:500, ABclonal Technology Co., Ltd., China, Cat # MM3373201) primary antibody was incubated for 12 h in 4°C, and goat anti rabbit IgG (H + L) PE fluorescent labeled second antibody (1:500, TransGen Biotech Co., Ltd, China, Cat # HS121-01) was incubated for 2 h in 4°C, DAPI was dripped after three times washing with PBS, and the stained tissue was observed and photographed under the microscope system (Cat #IX73, Olympus Corporation, Japan).

### Determination and Analysis of SCFAs Concentration in the Colonic Content

Fifty microliters of 15% phosphoric acid were added to 50 mg of colonic content, then 100 μL of 125μg/ml internal standard (isohexanoic acid) solution and 400 μL ether were added, tissue homogenate for 1 min, then centrifugation at 4° C and 12000 × g for 10 min. The supernatant was analyzed by gas chromatography-mass spectrometry (GC-MS, Agilent Technologies Inc., Agilent 6890N/5975B, USA). The column used was Agilent HP innowax capillary column (30 m × 0.25 mm ID × 0.25 μm) in the split injection mode. The injection volume was 1 μL and split ratio was 10:1. The temperatures of the injection port, ion source, line, and quadrupole were 250°C, 230°C, 250°C, and 150°C, respectively. The initial temperature of the programming was 90°C. The temperature was raised to 120°C (at 10 °C/min), followed by an increase to 150°C (at 5°C/min). Finally, the temperature was raised to 250°C for 2 min (at 25°C/min). The carrier gas was helium with a flow rate of 1.0 mL/min. The mass spectrometry used an electron impact ionization source and SIM scanning mode, and the electron energy was 70 ev (**Data Sheet 1**).

Based on the detection results, targeted quantification was carried out for the detected samples, and the relevant data were analyzed according to the quantitative results. According to the established sample pretreatment and instrumental analysis methods and standards, all samples were analyzed quantitatively. PCA was used to generate new characteristic variables by linear combination of metabolite variables according to a certain weight. The samples with poor repeatability (outlier samples) and abnormal samples were removed. The results of the orthogonal projections to latent structures discriminant analysis (OPLS-DA) can effectively reduce the complexity of the model and enhance the explanatory ability of the model without reducing the predictive ability of the model, so as to maximize the difference between groups. On this basis, the differences in metabolites between groups were analyzed to the maximum extent by using OPLS-DA.

SPSS19.0 was used to calculate the mean value and standard deviation of each group, and calculate the statistically significant P-value. Z-score (standard score) was calculated based on the content of metabolites, which is used to measure the content of metabolites at the same level, using the following formula: z = (x – μ)/σ (x is a specific fraction, μ is the average, σ is the standard deviation. The cor-function in R (v3.1.3) was used to calculate the correlation coefficient. When the linear relationship between the two metabolites was enhanced, the correlation coefficient tended to 1 or -1: positive correlation tended to 1, negative correlation tended to - 1. At the same time, the method of R is used cor.test *P≤ 0.05* had significant correlation.

### Microbial Genomic Sequencing

Total genomic DNA was extracted from the samples (n = 3) with a QIAamp DNA Stool Mini Kit (Qiagen, Hilden, Germany), according to the manufacturer’s instructions. DNA concentration and purity were evaluated on 1% agarose gel. The quantity of DNA was determined with a NanoDrop 1000 spectrophotometer (Thermo Fisher Scientific, USA) after calibration with sample solvents and the DNA was diluted to 1 ng/µL with sterile water. The V3–V4 distinct regions of the 16S rRNA genes were amplified with specific barcoded primers (32, 33). The PCR reactions were performed in triplicate in a total volume of 25 µL, consisting of 1 μL of each the primers (5 µM), 10 μL of 10 ng DNA template, 4 µL of 1× FastPfu buffer, 1 μL of 2.5 mM dNTPs, 0.4 µL FastPfu polymerase, and 7.6 μL nuclease-free water. The PCR program was as follows: initial denaturation at 94°C for 5 min, 30 cycles at 94°C for 50 s, 55°C for 30 s, 72°C for 50 s, and a final extension at 72°C for 6 min. The PCR products were purified with an AxyPrep DNA Gel Extraction Kit (Axygen Scientific Inc., USA). Amplicons from all samples were sent to a commercial company (Biomarker Technologies Corporation, China) for sequencing on an Illumina HiSeq 2500 platform (Illumina, USA). Sequence/expression data had been uploaded to NCBI database (PRJNA729581 and PRJNA744592 for heat stressed-pigs colonic microbiome project).

### Microbial Genomic Analyses

Species classification information corresponding to each operational taxonomic unit (OTU) was obtained by comparing the representative OTU sequences with the microbial reference database (SILVA, v. 138 and ITS, v 8.3). After rarefying OTU samples (RDP Classifier. 2.2; the confidence threshold is 0.8, sample size < minimum of samples (40000 reads) (34), sample community compositions were calculated at the phylum, class, order, family, genus, and species levels and generated in QIIME 2 (v 2021.2). GraphPad Prism v. 7.0 c (GraphPad Software, USA), R (v 3.1.3), Metastats, and STAMP (Statistical Analysis of Metagenomic Profiles) were used for the statistical analyses. The weighted UniFrac distances among the groups were statistically compared by analysis of similarities using the ‘vegan’ package of R (v 3.1.3). QIIME 2 software for Alpha diversity index of each sample, including the Shannon and Simpson indices. PERMANOVA/ANOSIM analysis is performed with binary jaccard (for unweightde unifrac) and bray Curtis, to cal (for weighted unifrac) culate the sample distance. We used R (v3.1.3) to draw rank abundance curves, species accumulation curves, PCA plots, and a heatmap of sample distances. PICRUST software was used to compare the species composition information obtained from 16S sequencing data to determine the functional gene composition of the samples (35), and the functional differences between different samples or groups were analyzed. Use PICRUST to compare the species composition information obtained by comparing 16S sequencing data, infer the functional gene composition in the sample, and analyze the functional differences between different samples or groups. In the univariate analysis of gut microbiota and predicted KEGG biochemical pathways for each group, a one-way ANOVA with Bonferroni’s multiple comparison test was performed to compare the diversities among the groups. Metastats identified differentially abundant phyla, genera, classes, and species in the groups. Significant differences between groups were identified by the LEfSe (Line Discriminant Analysis [LDA] Effect Size) method. At the genus level, the G-TEST and Fisher test were used to detect the differences in the abundance of species between samples. Pairwise t-tests were used to detect differences between groups, assuming a P-value threshold for significance of 0.05. RDA (Redundancy analysis) or CCA (Canonical Correspondence analysis) (36), distance-based redundancy analysis (db-RDA), and mantel test analysis in R language vegan package were used to analyze and map the relationships between the concentrations of SCFAs and serum biochemical indexes and changes in microbial flora.

### Transcriptome Sequencing and Analysis

In order to simplify the complexity of data analysis, we removed the time effect and only considered the influence of heat stress treatment on metabolism and intestinal changes. The enzymatic-free cryopreservation tube was pre-cooled in liquid nitrogen, and the tissue was quickly removed from the living body (cut into pieces sizes similar to soybean granules). Using RNase-free water to prepare 1× PBS or saline, the tissue surface stains were quickly cleaned, and the surface liquid was absorbed and collected into the freezing tube. Then, the freezer tube was rapidly put into liquid nitrogen for cryopreservation, and the samples were sent to the Sequencing Company (Majorbio, China) for sequencing. Eukaryotic mRNA sequencing is based on the HiSeq platform for sequencing all mRNAs transcribed from specific tissues of eukaryotes at a particular time. The total RNA was extracted from the tissue samples, the concentration and purity of the extracted RNA were detected by Nanodrop2000, the RNA integrity was detected by agarose gel electrophoresis, and the RIN value was determined by Agilent 2100. The eukaryotic mRNA 3’ end had a structure of a ployA tail, and the magnetic beads with Oligo (dT) were used for A-T base pairing with the flo A, and mRNA could be isolated from the total RNA for analysis of transcriptome information. The fragmentation buffer was added to randomly break the mRNA into small fragments of about 300 bp. Under the action of reverse transcriptase, six-base random hexamers were added, and mRNA was used as a template to reverse the synthesis of one-strand cDNA, followed by two-strand synthesis to form a stable double-stranded structure. After connecting to the adaptor, the short sequence fragments were sequenced using the Illumina HiSeq platform. Since the Illumina sequencing single-run can generate billions of reads, we used statistical methods to statistically quality-control the measured sequences by using Fastx toolkit (v 0.0.14, minimum sequence length: 30; minimum quality value: 20), which can visually reflect the library construction quality and sequencing quality of the samples. Hisat2 (v 2.2.1) was used for sequence alignment. And the quality data after the quality control (reads) was compared with the reference genome (Sus scrofa, v 11.1, Ensembl) to obtain the mapped data (reads) for subsequent analysis, and the quality of the comparison results of the sequencing is evaluated. New transcript function were noted by DIAMOND (v 0.8.37.99) for NR (NCBI non-redundant protein database) and Swiss-Prot.

Based on the existing reference genomes, the mapped reads were assembled and spliced by using software Cufflinks, compared with known transcripts, transcripts without annotation information, and functional annotations of potential new transcripts. Read Counts for each sample gene/transcript were obtained using RSEM (37). Using alignment to genome results and genome annotation files. This was then subjected to transcripts per million reads (TPM) conversion to obtain standardized gene/transcript expression levels by DESeq2. After obtaining the number of Read Counts of the gene/transcript, the differential analysis of the expression of the gene/transcript between the samples was performed on the multi-sample (≥2) project, and the differentially expressed genes/transcripts were identified to study the differentially expressed genes/transcription.

Weighted gene co-expression network analysis (WGCNA) was used to calculate the correlation coefficient between any two genes. To determine whether two genes have similar expression patterns, WGCNA analysis uses the weighted value of correlation coefficient, that is, the correlation coefficient of genes is N-th power, which makes the connection between genes in the network obey scale-free networks. Use the WGCNA package (v 1.46) for weighted correlation network analysis in R (v3.1.3). The WGCNA R software package is a comprehensive collection of R functions for performing various aspects of weighted correlation network analysis. The package includes functions for network construction, module detection, gene selection, calculations of topological properties, data simulation, visualization, and interfacing with external software. The SCFAs metabolite data and serum biochemical index data were introduced, and the hierarchical clustering tree was constructed by the correlation coefficient between genes. Genes with low expression or low coefficient of variation are generally considered noise, so the data for WGCNA needs to be pre-processed as much as possible to eliminate outliers. After data preprocessing, the genes/transcripts can be classified, and the expression patterns can be divided into modules. After obtaining the module, through the correlation analysis with the phenotypic data, the key module is obtained. Obtain the hub gene of the module through visualized network analysis. Different branches of the clustering tree represent different gene modules, and different colors represent different modules. Based on the weighted correlation coefficient of genes, genes are classified according to expression patterns, and genes with similar patterns are classified into a module. According to the module analysis, the correlation with environmental factors was analyzed, and the prediction network of gene/transcript interaction was drawn by Cytoscape (v. 3.6.0).

## Results

### Effects of HS on Blood Biochemical Indexes in Pigs

**Figure 1:**
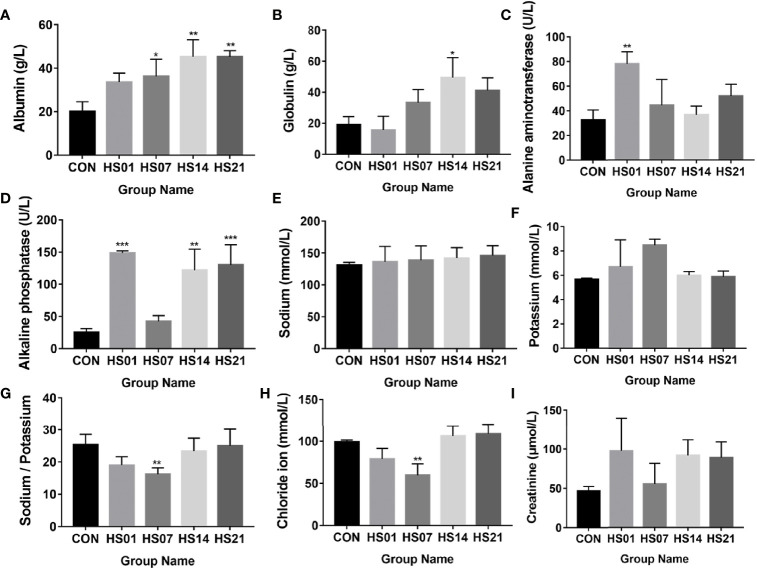
Effect of HS on the biochemical indexes of pig serum. CON indicates no heat stress group of day 1, HS1, 7, 14, 21 indicate heat stress day 1, 7, 14, and 21 groups respectively, n = 6; "*" indicates p < 0.05, "**" indicates p < 0.01, "***" means p < 0.001. **(A)** albumin; **(B)** globulin; **(C)** alanine aminotransferase; **(D)** alkaline phosphatase; **(E)** sodium ion; **(F)** calcium ion; **(G)** calcium to sodium ratio; **(H)** chloride ion; **(I)** creatinine.

The concentrations of serum albumin and globulin gradually increased with the extension of HS compared with that in control, and showed the significance in day 14 of heat stressed pigs (*P <* 0.05) (**Figures 1A, B**). Alanine aminotransferase level was significantly (*P <* 0.01) higher on day 1 of HS than that in the control group (**Figure 1C**). Compared with the control group, alkaline phosphatase was significantly increased (*P <* 0.01) after HS treatment on days 1, 14, and 21 of HS, but no show the difference between the control and the day 7 of HS (**Figure 1D**). As shown in **Figures 1E–G**, there was no significant change in serum calcium and sodium after HS treatment, but the ratio of calcium and sodium decreased significantly compared with that in the control group on day 7 of HS. While compared with the control, the level of chloride ion and creatinine were decreased on day 7 of HS, but it also showing the rising trend on days 1, 14, and 21 of HS (**Figures 1H, I**).

**Figure 2:**
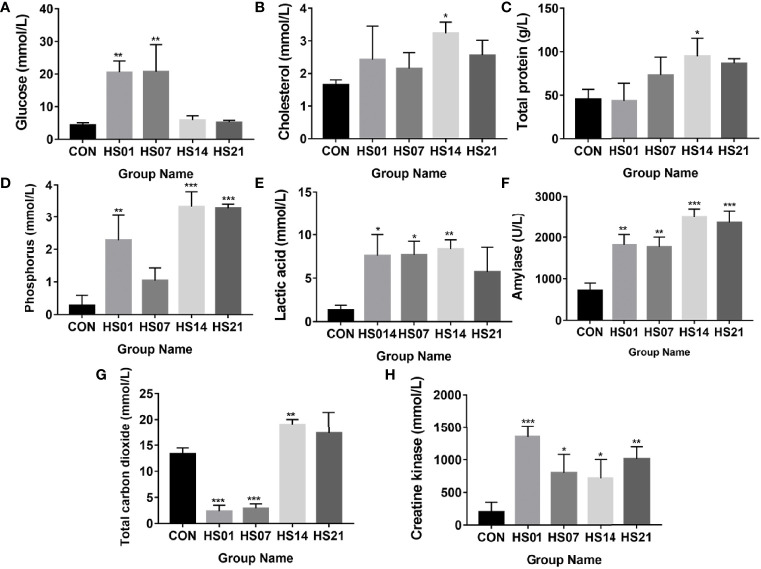
Effect of HS on biochemical indexes in pig serum. CON indicates no heat stress group of day 1, HS1, 7, 14, 21 indicate heat stress day 1, 7, 14, and 21 groups respectively, n = 6; “*” indicates p < 0.05, “**” indicates p < 0.01, “***” means p < 0.001. **(A)** glucose; **(B)** cholesterol; **(C)** total protein; **(D)** inorganic phosphorus; **(E)** lactic acid; **(F)** amylase; **(G)** total carbon dioxide; **(H)** creatine kinase.

Compared with the control group, the concentration of glucose was significantly increased (*P <* 0.01) on days 1 and 7 of HS (**Figure 2A**), while the concentration of cholesterol and total protein were significantly increased (*P <* 0.05) on day 14 of HS (**Figures 2B, C**). The concentration of inorganic phosphorus was significantly higher (*P <* 0.01) than that in control group on days 1, 14, and 21 of HS (**Figure 2D**), but no show the difference between the control and the day 7 of HS. The levels of lactate and amylase in the HS group were higher than that in the control group (**Figures 2E, F**). Compared with the control group, the total carbon dioxide was significantly decreased (*P <* 0.001) on days 1 and 7 of HS and increased significantly (*P <* 0.01) on day 14. The concentration of creatine kinase was significantly increased (*P <* 0.05) in the HS group compared with that in the control (**Figures 2G, H**).

**Figure 3:**
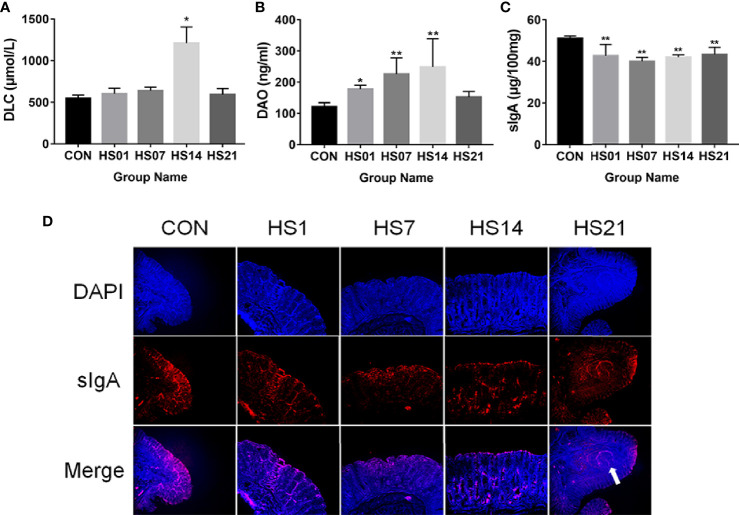
Effect of HS on the biochemical markers of serum and colon. CON indicates no heat stress group of day 1, HS1, 7, 14, 21 indicate heat stress day 1, 7, 14, and 21 groups respectively, n = 6; “*” indicates p < 0.05, and “**” indicates p < 0.01. **(A)** serum D-lactic acid; **(B)** serum diamine oxidase; **(C)** sIgA of colonic content; **(D)** distribution of sIgA in colon.

The D-lactic acid was only showing the significantly (*P* < 0.05) increased on day 14 of HS, but don ‘t have difference on days 1, 7, and 21, by comparing with the control group (**Figure 3A**). Compared with the control group, the concentrations of serum diamine oxidase were significantly increased on days 7 and 14 of HS (*P <* 0.01), peaked at day 14, and decreased at day 21 (**Figure 3B**). Compared with the control group, HS treatment significantly reduced (*P <* 0.01) the level of Secretory immunoglobulin A (sIgA) in the colonic content (**Figure 3C**). And as showing in **Figure 3D**, the sIgA was mainly located in the intestinal epithelium and lymph nodes (as the arrow mentioned) of the colon.

### Effect of HS on the Morphological Structure of Pig Colon

**Figure 4:**
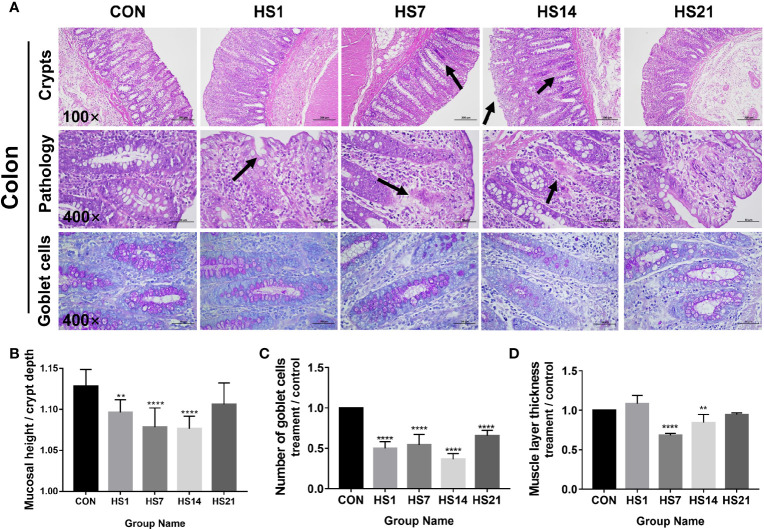
Effect of HS on the morphology structure of colon. CON indicates no heat stress group, HS1, 7, 14, 21 indicate heat stress day 1, 7, 14, and 21 groups respectively, n = 6; “**” indicates p < 0.01, “****” means p < 0.0001. **(A)** HE and PAS staining results of paraffin sections of colon; **(B)** villus height/crypt depth; **(C)** PAS positive cell number; **(D)** intestinal muscle layer thickness. As the arrows showing, after HS treatment, the colonic mucosal were loosely arranged and showed signs of bleeding on days 1, 7 and 14 of HS, the local injury and lymphatic infiltration also had been found on days 7 and 14 of HS.

After HS treatment, the colonic mucosal were loosely arranged and showed signs of bleeding on days 1, 7 and 14 of HS, the local injury and lymphatic infiltration also had been found on days 7 and 14 of HS (**Figure 4A**). The intestinal lymph nodes were enlarged on day 14 of HS (**Figure 4A**). The mucosal height/crypt depth of colon was significantly decreased *(P <* 0.001) on days 1, 7, and 14 of HS (**Figure 4B**). Periodic acid-Schiff (PAS)-positive cells in the colon of HS pigs were significantly decreased (*P <* 0.0001) than those in the control group (**Figure 4C**). The thickness of the muscle layer decreased on days 7 and 14 of HS compared with that in the control group (**Figure 4D**).

### Effect of HS on the Metabolism of SCFAs in Pig Colon

**Figure 5:**
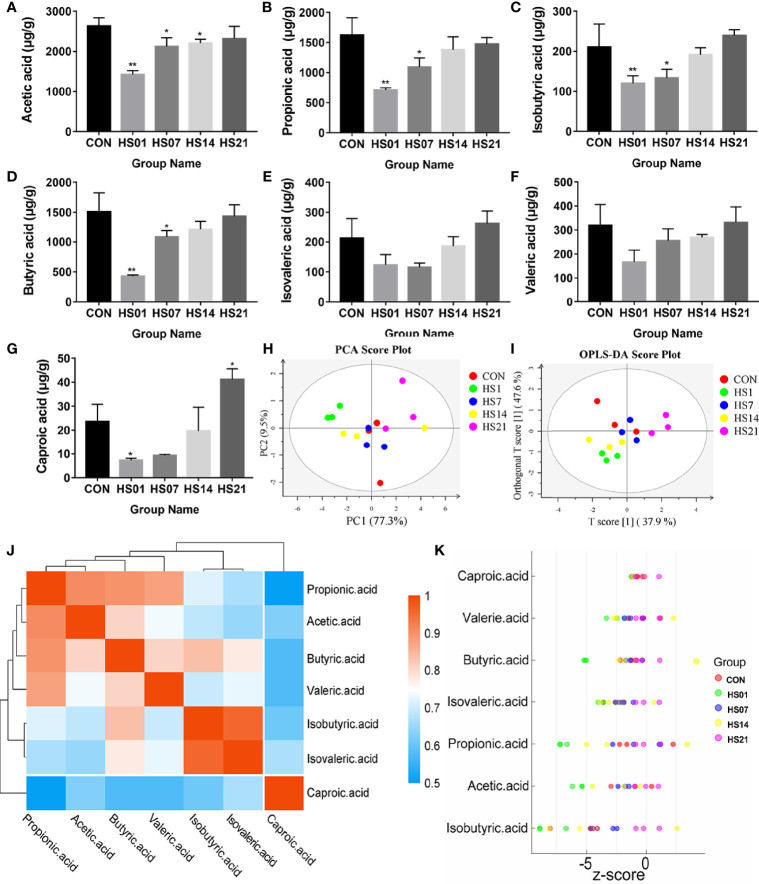
Effect of HS on the metabolism of SCFAs in pig colon. CON indicates no heat stress group of day 1, HS1, 7, 14, 21 indicate heat stress day 1, 7, 14, and 21 groups respectively, n = 4; “*” indicates p < 0.05, and “**” indicates p < 0.01. **(A)** acetic acid; **(B)** propionic acid; **(C)** isobutyric acid; **(D)** butyric acid; **(E)** isovaleric acid; **(F)** valeric acid; **(G)** caproic acid; **(H)** PCA analysis between short-chain fatty acid samples; **(I)** short-chain OPLS-DA analysis between fatty acid samples; **(J)** metabolite correlation analysis; **(K)** Z-score analysis.

After analysis, we found that the microbes of the control group were not significantly different at different time points. Subsequently, we used SCFAs metabolome and transcriptome tests to analyze whether microbial changes can cause the activation of intestinal inflammation pathways through SCFAs. In order to simplify the complexity of data analysis, we removed the time effect and only considered the influence of heat stress treatment on metabolism and intestinal changes. As shown in **Figure 5**, the concentrations of acetic acid, propionic acid, butyric acid, isobutyric acid, and caproic acid were significantly reduced (*P <* 0.05) in the HS group compared with that in the control groups, and gradually reached control levels by day 21; and the valeric and isovaleric acids were shown the same trend but not significant (**Figures 5A–G**). The metabolite variables were grouped by linear combination according to certain weight, and the data of each group were classified by main new variables (principal component analysis [PCA]), and samples with poor repeatability and abnormal samples were removed. As a result, all samples had no abnormal outliers, among which the samples on days 1 and 14 of HS were far away from the control group (**Figure 5H**). The results of the orthogonal projections to latent structures discriminant analysis (OPLS-DA) can effectively reduce the complexity of the model and enhance the explanatory ability of the model without reducing the predictive ability of the model, so as to maximize the difference between groups. OPLS-DA showed that there was significant difference between the HS group and the control group, except on day 7 of HS (**Figure 5I**). The results of metabolic difference analysis between SCFAs showed that there was a strong positive correlation (correlation coefficient more tended to 1) between acetic acid, propionic acid, butyric acid and valeric acid, and a positive correlation was observed between isobutyric acid and isovaleric acid (**Figure 5J**). Z-score (standard score) was calculated based on the content of metabolites, which is used to measure the content of metabolites at the same level. The results of Z-score analysis showed that the metabolite content in the HS group on days 1 and 14 was significantly lower than that in the control group, and the content of SCFA metabolites in the colons of pigs on day 21 was slightly higher than that in the control group (**Figure 5K**).

### Effect of HS on the Microbiome of Pig Colon

**Figure 6:**
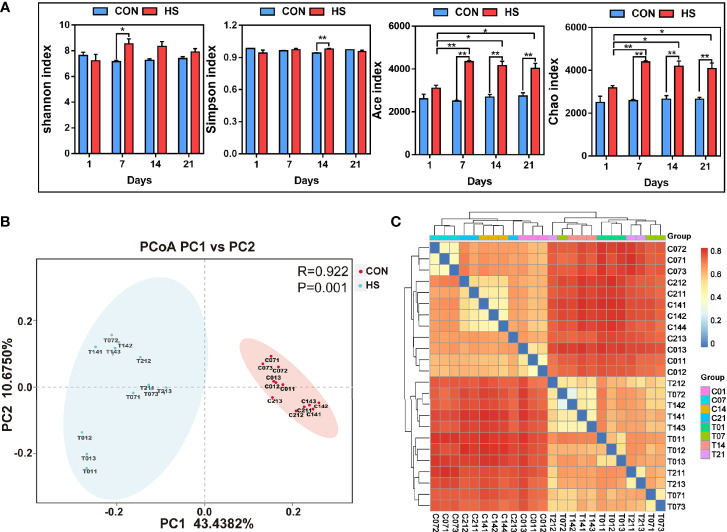
The effect of heat stress on the microbial diversity of pig colon. CON and C indicate no heat stress group, HS and T indicate heat stress treatment, 1, 7, 14, 21 indicate day 1, 7, 14, and 21 groups respectively, n = 3; “*” indicates p < 0.05, and “**” indicates p < 0.01. **(A)** inter-group Alpha diversity analysis of Shannon index, Simpson index, Ace index and Chao Index; **(B)** principal component analysis between samples; **(C)** species composition heat map between samples.

The results of alpha diversity index showed that the homogeneity of samples in each group was high, and the Shannon index in day 7 of HS group was significantly (*P* < 0.05) lower than that in control groups, while Simpson index in day 14 of HS group was significantly (*P* < 0.01) lower than that in day 14 of control groups. The Chao and Ace indexes in day 7, 14 and 21 of HS group was significantly (*P* < 0.01) higher than that in each control groups (**Figure 6A**). PCA analysis showed that there was a significant difference in the flora of the HS and control groups (**Figure 6B**). The cluster thermogram (**Figure 6C**) showed that the distance between HS groups in different time was closer, and the cluster relationship between the control groups in different time was closer.

**Figure 7:**
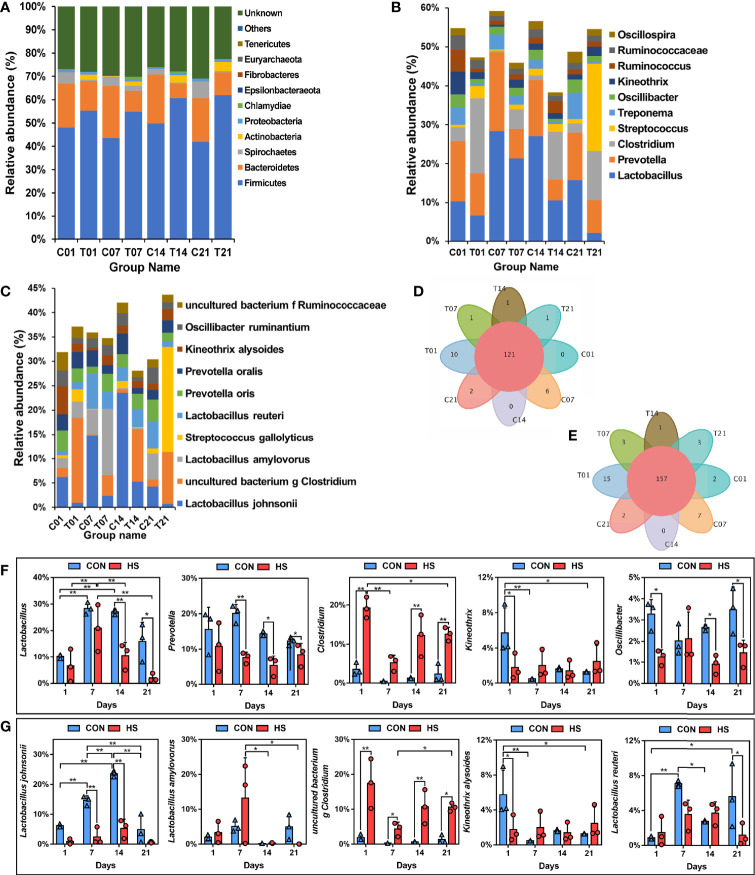
Effect of HS on the microbial structure of pig colon. CON and C indicate no heat stress group, HS and T indicate heat stress treatment, 1, 7, 14, 21 indicate day 1, 7, 14, and 21 groups respectively, n = 3; “*” indicates p < 0.05, “**” indicates p < 0.01. **(A)** TOP10 by phylum level; **(B)** TOP15 by genus level; **(C)** TOP15 by species level; **(D)** Venn plot of genus level; **(E)** Venn plot of species level; **(F)** differences of phylum level by TOP5; **(G)** differences of genus level by TOP5.

At the phylum level, all groups contained ten phylum level microorganisms, namely Firmicutes, Bacteroidetes, Spirochaetes, Actinobacteria, Proteobacteria, Chlamydiae, Patescibacteria, Tenericutes, Cyanobacteria, and Euryarchaeota; Among them, Firmicutes and Bacteroides were predominant, and their total number accounts for more than 60% (**Figure 7A**). Venn analysis showed that there was among the same 121 genera in each group (**Figure 7D**). The top 5 bacteria by genus level in the total distribution were Lactobacillus (the largest proportion is greater than 30%), Prevotella (2 – 20%), Clostridium (the largest proportion is greater than 27%), Streptococcus (2 – 20%), and Treponema (0 – 11%, **Figure 7B**). The bacteria in the first 5 bacteria genus levels had a relatively high abundance of 5% were observed as shown (**Figure 7F**). The percentage of Lactobacillus decreased significantly (decreased by more than 16% and 14% by compared with each control group) on the days 14 and 21 of HS, as well as the Prevotella by compared with each control group (decreased by 12%, 9% and 3% respectively on day 7, 14 and 21, P < 0.05); whereas the percentage of Clostridium increased by more than 11% after HS treatment on day 14 and 21. Kineothrix decreased significantly (more than 4%, P < 0.05) on day 1 of HS, and Oscillibacter also decreased significantly (more than 2%, P < 0.05) on days 1, 14 and 21 of HS.

Venn analysis also showed that there was among the same 157 species in each group, and day 1 of HS possessed 15 unique species (**Figure 7E**). The top 5 bacteria by species level in the total distribution were *Lactobacillus johnsonii, uncultured bacterium g Clostridium, Lactobacillus amylovorus, Streptococcus gallolyticus*, and *Lactobacillus reuteri* (**Figure 7C**). The bacteria in the first 5 bacteria species levels had a relatively high abundance of 5% were observed as shown (**Figure 7G**). The percentage of *Lactobacillus johnsonii* was decreased significantly (*P < 0.01*) on the days 7 and 14 of HS, 12.4% and 18.3% respectively, by compared with each control group, *Lactobacillus amylovorus* was increased 8.63% in day 7 of HS; whereas the percentage of *uncultured bacterium g Clostridium* increased after HS treatment (increases by 15.5%, 4.0%, 10.1%, and 9.2% respectively, *P <* 0.05). *Kineothrix alysoides* decreased significantly (4.00%, *P* < 0.05) on day 1 of HS, and *Lactobacillus reuteri* also decreased significantly (4.5%, *P* < 0.05) on day 21 of HS.

**Figure 8:**
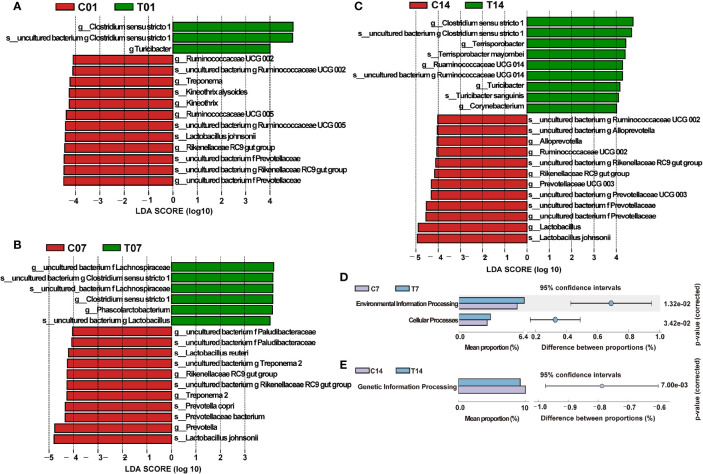
Effect of HS on the difference in microbial composition of pig colon. CON and C indicate no heat stress group, HS and T indicate heat stress treatment, 1, 7, 14, 21 indicate day 1, 7, 14, and 21 groups respectively; **(A)** LEfSe analysis between C01 and T01 groups; **(B)** LEfSe analysis between C07 and T07 groups; **(C)** LEfSe analysis between C14 and T14 groups; **(D, E)** KEGG metabolism prediction differences between groups in day 7 and 14 of HS.

We compared the differences of dominant bacteria (genus and species) between HS group and control group on the days 1, 7 and 14 (**Figure 8A**). The dominant bacteria in the control group of day 1 were *Lactobacillales johnsonii*, *Rikenellaceae RC9 gut group, Kineothrix alysoides* and *Prevotellaceae*, etc; in the control group of day 7, dominant bacteria were *Lactobacillus reuteri, Prevotella copri, Rikenellaceae RC9 gut group, Lactobacillales johnsonii* and *Treponema 2*, etc.; While the dominant bacteria in the control group of day 14, also had *Prevotellaceae*, *Lactobacillales johnsonii* and *Rikenellaceae RC9 gut group*. In contrast, the dominant bacteria on days 1, 7 and 14 of HS group were *Clostridium sensu stricto 1*, while on day 14, the dominant bacteria of HS group also had *Terrisporobacter* and *Turicibacter sanguinis*, etc., indicating that HS could significantly change the microbial structure and increase the content of opportunistic pathogens (**Figures 8B, C**). The KEGG difference prediction analysis based on PICRUST found that the colonic microbiota may have an impact on Environmental information processing and Cellular processes in pigs on the day 7 of HS by compared with its’ control group (*P <* 0.01, **Figure 8D**). And it also shows that colonic microbiota may affect pigs’ genetic information processing in day 14 of HS (*P <* 0.01, **Figure 8E**).

**Figure 9:**
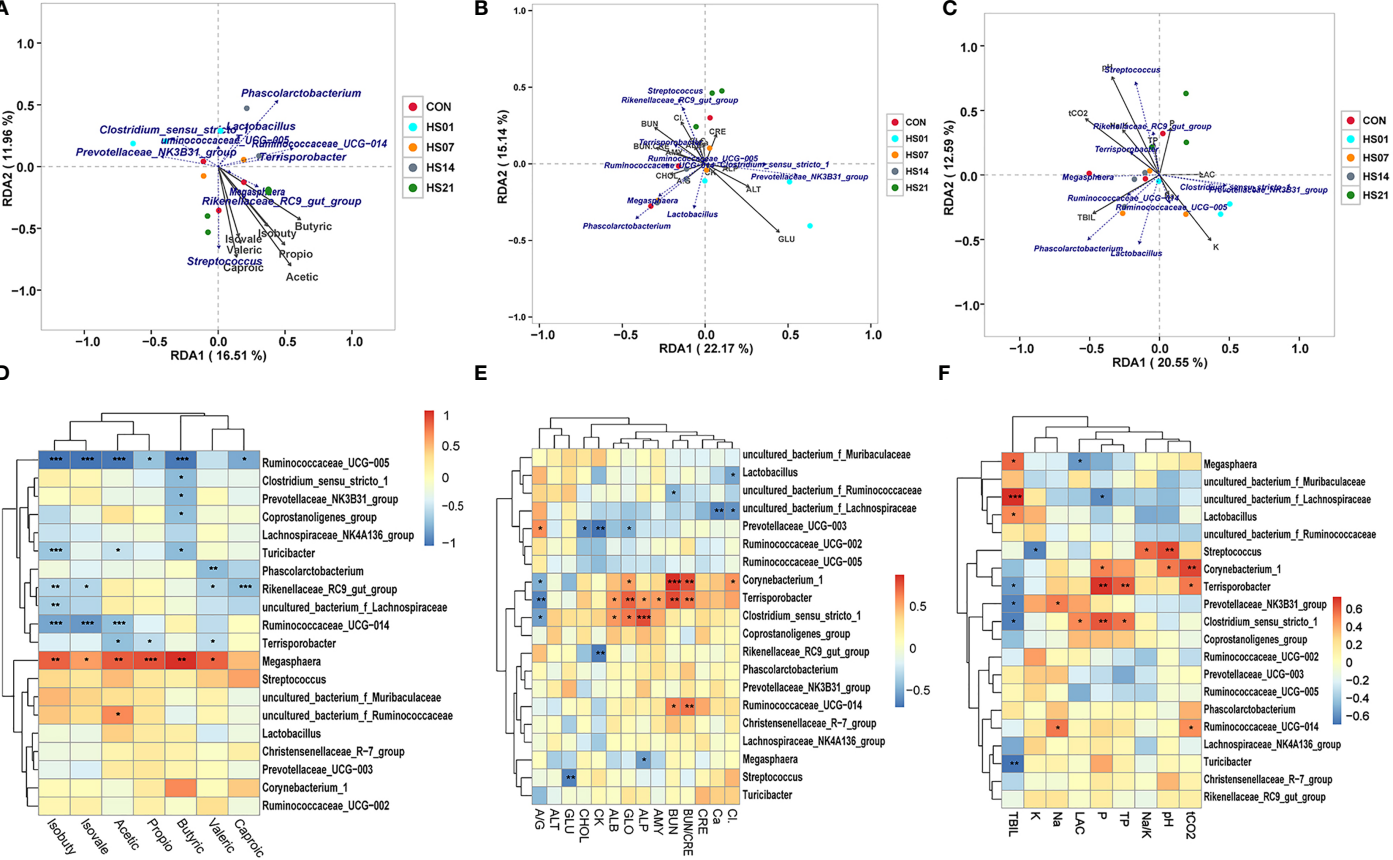
Correlation analysis of microbiome and metabolites. “*” means p < 0.05, “**” means p < 0.01, “***” means p < 0.001; **(A–C)** RDA/CCA correlation analysis of metabolites and microorganisms; **(D–F)** heat map of metabolites and microbiotas correlation. Sorry, I don’t know how to analyze this, can you provide the corresponding references.

Redundancy analysis/canonical correspondence analysis (RDA/CCA) were used to analyze the correlation among microbiome and SCFAs metabolites in each group. As shown in **Figure 9A**, the cumulative contribution rate of SCFAs to the structure of the flora was 9.78% and 15.39%, respectively. We found that the abundance of *Turicibacter sangunis* was negatively correlated with SCFAs metabolism, whereas the changes in the abundance of *Megasphaera cf* was positively correlated with SCFAs metabolism. In addition, the abundance of *Terrisporobacter mayombei, Lactobacillus reuteri, lactobacterium succinatutens* were negatively correlated with isobutyric acid, valeric and caproic, but positive correlated with butyric. There was a positive correlation between the metabolism of isobutyric acid, valeric and caproic and that of *Kineothrix alysoides, Treponema procinum* and *Lactobacillus amylovorus*. In addition, there was a positive correlation between *Megasphaera cf* and SCFAs metabolism, indicating the beneficial role of *Megasphaera cf* in fatty acid metabolism. In contrast, there was a negative correlation between *Ruminococcaceae* and SCFAs, except propionic acid, indicating its negative regulatory role in SCFAs metabolism. In addition, in terms of flora composition and SCFAs metabolism composition, there was a pairwise comparison of the negative correlation between isobutyric acid, isovaleric acid and *Megasphaera cf*, *Turicibacter sanguinis*, *Terrisporobacter mayombei*, and *Ruminococcaceae UCG−005* (**Figures 9A, D**). As shown in **Figures 9C–E**, RDA showed that the cumulative contribution rate of serum biochemical index to the structure of the flora was 13.53% and 22.36%, 9.8% and 15.37%, respectively. The changes in the percentage of *Lactobacillus reuteri*, *Megasphaera cf*, *Phascolarctobacterium succinatutens* and *Lactobacillus johnsonii* were positively correlated with the changes in serum total bilirubin and the ratio of albumin and globulin, while were negatively correlated with the changes in serum creatinine, alanine transaminase, potassium ion, alkaline phosphatase and albumin. The changes in the percentage of *Lactobacillus amylovorus, Kineothrix alysoides, Turicibacter sanguins and Treponema porcinum* were positively correlated with the changes in serum chloride, alkaline phosphatase, potassium ion, alanine transaminase and creatinine, while were negatively correlated with the changes in serum globulin, total carbon dioxide, total bilirubin and the ratio of albumin and globulin (**Figure 9B**). Besides, abundance of *Lactobacillus reuteri, Megasphaera cf, and Lactobacillus amylovorus* have negative correlation with calcium ions, lactic acid, creatine kinase, amylase, inorganic phosphorus and total bilirubin; the abundance of *Lactobacillus johnsonii, Treponema porcinum*, *Phascolarctobacterium succinatutens* and *Terrisporobacter mayorrbei* have negative correlation with serum pH, total protein, total cholesterol; while negatively correlated with glucose, lactate and creatine kinase. The concentrations of serum glucose was positively correlated with the *Kineothrix alysoides*. The abundance of *Turricibacter sanguinis* was positively correlated with lactic acid, creatine kinase, amylase, inorganic phosphorus (**Figure 9C**). Correlation heat map analysis found that *Lactobacillus amyovorus* has a negative correlation with liver and kidney metabolism in pigs, and *Ruminococcaoeae UCG-014* have a negaitive correlation with liver and kidney metabolism in contrast. While *Clostridium sensu stricto 1* has a positive correlation with alanine aminotransferase, alkaline phosphatase, creatinine, amylase, inorganic phosphorus, lactic acid, creatine kinase and total cholesterol (**Figures 9E, F**).

As shown in **Table 1**, Mantel-test showed that acetic acid is the key (R = 0.200197, *P* = 0.043) to determine the microbial composition, whereas isobutyric acid and isovaleric acid were negatively correlated with the microbial composition. With respect to the biochemical indexes, the concentrations of lactic acid, amylase, creatine kinase, and potassium ion, and pH of the serum were positively correlated with the changes in colonic flora structure (*P <* 0.05).

**Table 1:** Correlation analysis of microorganisms and metabolites Mantel-test.

Environmental factors	Mantel-test R statistic	p-Value	Environmental factors	Mantel-test R statistic	p-Value
Acetic	0.200197	0.043	Ca^2+^	0.040517	0.349
Propionic	0.146603	0.102	Na^+^	-0.00882	0.504
Isobutyric	-0.07419	0.722	K^+^	0.245255	0.023
Butyric	0.149879	0.108	Na^+^/K^+^	0.172682	0.077
Isovaleric	-0.10435	0.822	BUN	-0.07445	0.754
Valeric	0.086125	0.213	CRE	-0.02208	0.564
Caproic	0.109714	0.209	BUN/CRE	-0.17751	0.938
GLU	0.170201	0.06	TBIL	0.085839	0.232
AMY	0.20936	0.044	tCO_2_	0.096218	0.15
CHOL	0.121927	0.168	CK	0.225902	0.032
LAC	0.213002	0.048	ALP	0.159538	0.073
pH	0.245673	0.021	ALT	0.120687	0.189
P	0.07994	0.2	ALB	0.022509	0.409
Cl^-^	0.138532	0.123	GLO	-0.05131	0.687
TP	-0.01936	0.545	A/G	-0.05389	0.665

### Transcription Sequencing Analysis of Colon Tissue in HS Pigs

**Figure 10:**
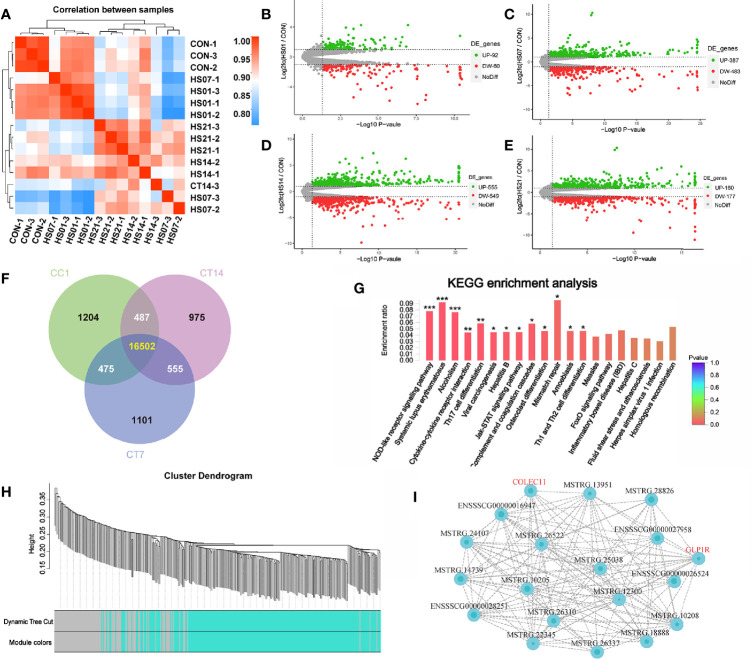
Transcription sequencing analysis of HS pig colon. CON indicates no heat stress group of day 1, HS1, 7, 14, 21 indicate heat stress day 1, 7, 14, and 21 groups, respectively, n = 3. “*” means p<0.05, “**” means p<0.01, “***” means p<0.001; **(A)** distance heat map between samples; **(B-E)** expression difference volcano plots. **(F)** Venn analysis between groups, **(G)** difference gene KEGG enrichment analysis; **(H)** WGCNA modular analysis; **(I)** WGCNA module related gene interaction network of green module.

From the sequencing results, we found that the correlation heat map between the samples showed that the distances of samples in HS groups were close (**Figure 10A**). Based on the differential expression volcanic plots, we found that when the difference threshold (HS/control) was 2, on days 1, 7, 14, and 21, 92, 387, 555, and 461 genes were upregulated (*P <* 0.05), respectively, while 60, 483, 549, and 467 genes were down-regulated, respectively (**Figures 10B–E**).

Venn analysis of the genes in the control group and HS groups on days 7 and 14 showed that there were 555 genes that significantly changed in HS pigs (**Figure 10F**). These genes were enriched in the immune-related signaling pathways, such as the nucleotide-binding oligomerization domain (NOD)-like receptor signaling pathway, systemic lupus erythematosus, cytokine and cytokine receptor interaction, Th17 cell differentiation signaling pathway, janus kinase (JAK)- signal transducer and activator of transcription (STAT) signaling pathway, Th1 and Th2 cell differentiation, the forkhead box O signaling pathway, and IBD signaling pathway. In addition, related functions such as Alcoholism Viral carcinogenesis, Hepatitis B, Mismatch repair and Amoebiasis pathways were also enriched by analysis (**Figure 10G**).

After a co-expression weighted association analysis for the 555 genes (transcripts) and SCFAs, we found that 327 genes (transcripts) were associated with SCFAs metabolism. Based on the modules in blue, we constructed an interaction network of pathways using data from GO and KEGG database, and the 152 genes were annotated. Specifically, the gray module was utilized for housing the genes that were not co-expressed with other genes and thus could not be assigned to any of the other modules; we have ignored these in in our study. In addition, according to the green module, a total of 41 known genes were significantly related to the metabolites of SCFAs. Among the green module genes, glucagon like peptide receptor (GLP1R) and collagen lectin family protein 11 were significantly associated with SCFAs metabolism (**Figures 10H–I**).

**Figure 11:**
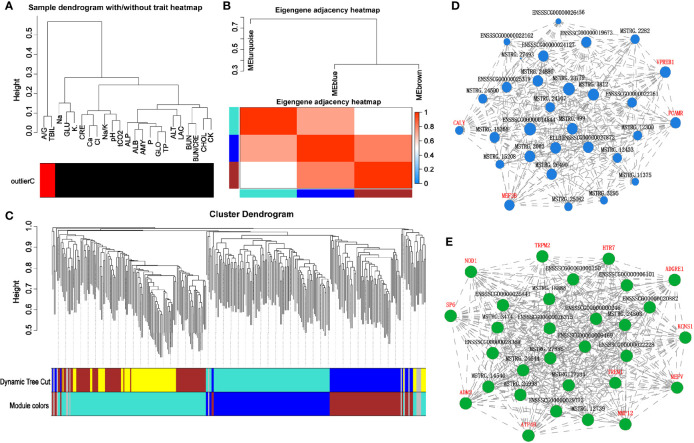
Correlation analysis of heat-stressed pig colonic transcriptome and serum biochemical indicators. **(A)** WGCNA cluster analysis; **(B, C)** WGCNA modular analysis; **(D, E)** WGCNA module related gene interaction network.

Using the WGCNA analysis for the correlation between serum biochemical indexes and the above 555 genes (transcripts), we first eliminated the outlier indexes, total bilirubin and A/G, as they had no correlation with the transcripts (**Figure 11A**). Next, the significant changed genes (transcripts) in the HS group on days 7 and 14 were divided into four modules, according to their correlation trend (**Figures 11B, C**). Based on these three modules‐green, tan, and blue, (the gray module was utilized for housing the genes that were not co-expressed with other genes, we have ignored these in study.) we constructed an interaction network of pathways using data from GO and KEGG database. The blue module could recognize the interaction network of neuron-specific vesicular protein calcyon, testis-specific serine kinase, cellular retinoic acid-binding protein 1, sialoadhesin, and serum biochemical indicators (**Figure 11D**). The green module could recognize matrix metalloproteinase-12, cell adhesion molecule 2, adhesion G protein coupled receptor E1, triggering receptor expressed on myeloid cells 2, transcription factor 6, potassium voltage-gated channel delayed-rectifier subfamily S member 1, hydrogen potassium exchange ATP and subunit β, and the interaction network of *ATP4B* and other genes with serum biochemical indicators (**Figure 11E**).

## Discussion

When animals are exposed to high temperature environment, water loss by evaporation and heat dissipation of the body through the epidermis is increased, which may lead to electrolyte imbalance (38–40). This in turn may decrease the blood sodium and chloride ion concentration (41, 42), and thereby affect the functioning of the urinary system. Furthermore, water loss may lead to dehydration, affecting the normal function of the kidney (43). In this study, the total protein, albumin, and the concentration of serum creatinine were increased in HS pigs, indicating that electrolyte balance and renal function were affected by HS. Furthermore, some biochemical indexes related to liver function, such as globulin, alanine aminotransferase, and cholesterol, also increased in HS pigs, suggesting that the liver metabolic function changes under HS. The interaction between calcium and chloride ions has been reported to affect the intestinal barrier function and transportation of nutrients (44, 45).

Alkaline phosphatase not only plays an important role in the absorption of calcium, fatty acids, and other minerals or nutrients, but also can prevents the invade of bacterial components Lipopolysaccharide (LPS) and flagellin (46–48). When alkaline phosphatase binds to intestinal epithelial cells, it inhibits the growth of potential pathogens, such as *Escherichia coli* (49). In addition, alkaline phosphatase and the secreted heat shock proteins participate in the colonization of intestinal microorganisms in piglets and play an important role in the production of antioxidant enzymes and anti-inflammatory and regulation of the intestinal function (46, 50, 51). In this study, alkaline phosphatase increased significantly after HS treatment. We hypothesized that the damage to intestinal balance induced the regulatory function of host and resulted in the upregulation of alkaline phosphatase.

Diamine oxidase and D-lactic acid are used as clinical indicators of enteritis, intestinal tissue, and barrier damage (52, 53). We found that both diamine oxidase and D-lactic acid increased in HS pigs, indicating that the occurrence of intestinal damage. Analysis of the morphological structure showed that villus height/crypt depth decreased significantly in the gut of HS pigs, and the thickness of intestinal muscle layer decreased. Under normal physiological conditions, the mucous layer covers the microvilli of epithelial cells, which is the first protective barrier to prevent intestinal microorganisms from entering the mucosal layer (54–56). Goblet cells are the main source of mucus, and a decrease in goblet cells has been reported to result in the invasion of bacteria (56). sIgA is an important physical barrier in the intestine. It can inhibit the adhesion and colonization or invasion of pathogenic microorganisms to the surface of intestinal mucosa (20). In HS pigs, sIgA was significantly reduced in both colon tissues and contents, suggesting a serious intestinal barrier injury.

Intestinal microflora participate in the digestion of carbohydrates and produce SCFAs, which protect the epithelium from injury, participate in the synthesis of essential amino acids, regulate lipid metabolism, induce intestinal peristalsis, improve and regulate intestinal angiogenesis, and activate of the immune system (57, 58). In addition, SCFAs regulate the production, transportation, and function of innate and adaptive immune cells. Butyric acid is a type of SFA that can directly act on the immune cells in the intestinal mucosa, increase the number and activity of Treg, and inhibit the activity of neutrophils, macrophages, dendritic cells, and effector T cells (59). It is commonly used as a marker of intestinal homeostasis (60). We found that under HS, the content of butyric acid in colonic content decreased significantly, in addition to the decrease in acetic acid, propionic acid, and hexanoic acid, thus indicating that HS affected the metabolism of SCFAs in pigs.

The dominant bacteria in the control group were beneficial bacteria, such as *Lactobacillus.* while the dominant bacteria in HS group were pathogenic bacteria, such as *Clostridium sensu stricto 1* and *Terrisporobacter*, which play a vital role in the occurrence of intestinal inflammation (61–63). Correlation analysis with serum biochemical indexes showed that *Phascolarctobacterium*, *Lactobacillus* and *Megasphaera cf* were positively correlated with the concentrations of serum cholesterol, creatine kinase, and total bilirubin, while negatively correlated with the concentrations of lactic acid, creatinine, sodium ion, albumin, globulin, and alkaline phosphatase, thus suggesting that intestinal beneficial bacteria can maintain the health of the pigs under normal condition. *Clostridium sensu stricto 1* is an opportunistic pathogen (63, 64), which can cause intestinal inflammation and decrease the content of SCFAs (65). In our study, we found that this pathogen was significantly increased on day 1 of HS, and was negatively correlated with serum total bilirubin, and positively correlated with the content of lactic acid, inorganic phosphorus, total protein, globulin, albumin, and alkaline phosphatase, thus indicating that it may be involved in the occurrence of intestinal inflammation in HS-pigs.

The level of SCFAs and microbial structure were correlated closely (66). In this study, the cumulative contribution rate of SCFAs to the structure of the flora was 9.78% and 15.39%, respectively. We found that the abundance of Turicibacter sangunis was negatively correlated with SCFAs metabolism. Although *Megasphaera cf* was positively correlated with SCFAs metabolism, *Clostridium sensu stricto 1* was negatively correlated with SCFAs except propionic acid. We hypothesized that the change in dominant bacteria could change the metabolism level of SCFAs. Gonçalves et al. found that there is a positive regulatory relationship between the metabolism of *Phascolarctobacterium* and *Lactobacillus* and the metabolism of acetic acid and butyrate (59), which is consistent with the findings of our study.

HS can significantly change the expression of multiple genes (transcripts), which are enriched in many inflammatory immune related signaling pathways, such as toll like receptor signal, interleukin (IL)-17 signal, NOD-like receptor signal, and JAK-STAT signal. In metabolite transcriptome association analysis, glucagon like peptide receptor (GLP1R), which is related to SCFAs metabolism, can activate nuclear factor kappa light chain enhancer of activated B cells signaling pathway (67) and Myosin light-chain (68) *via* cAMP, leading to inflammation and intestinal barrier damage, indicating that the metabolism of SCFAs is related to HS-induced intestinal inflammation. This finding can be considered as a breakthrough point for further studies on the pathogenesis of IBD in pig.

In this study, we ignored the time effect in the setting of the control group (**Supplementary Material 2**), only designed the heat stress effect in bacteria diversity, SCFA, and transcriptome. We found that the structure, diversity, and metabolite levels of intestinal microorganisms in HS-pigs changed significantly, which led to the activation of immune response and inflammation signal pathways and caused abnormal physiological and biochemical indexes and intestinal mucosal damage in pigs. The results provide useful information to understand the important of intestinal flora in stress induced IBD and suggest that maintenance of intestinal flora balance may be a useful technique for IBD therapy.

## Health and Safety

All our experimental procedures were conducted according to the universally accepted codes of laboratory practice and/or as per manufacturer’s instructions and followed all health and safety guidelines advocated by them.

## Data Availability Statement

The datasets used and/or analyzed during the current study are available from the corresponding author upon reasonable request.

## Ethics Statement

The animal study was reviewed and approved by Animal Care and Use Committee of Guangdong Ocean University, Zhanjiang, China (Permit No. 206-1108).

## Author Contributions

Author order was determined both alphabetically and in order of increasing seniority. XJ conceived the project and designed the experiments. CH, XN, SC, JW, MB, SM, and LW conducted the experimental work and analyzed the data. XJ interpreted the results. CH prepared the figures and wrote the manuscript. XJ and YY edited the manuscript. XM, XL and ZY participated in the enrichment analysis, manuscript writing and revision. All authors contributed to the article and approved the submitted version.

## Funding

This study was supported by the National Natural Science Foundation of China [grant numbers 31472243, 31902314]; Natural Science Foundation of Guangdong Province, China [grant number: 2019A1515011142]; the Project of Enhancing School with Innovation of Guangdong Ocean University [grant number: GDOU230419057]; the Basic Research Project of Shenzhen Science and Technology Innovation Commission (JCYJ20190813142005766).

## Conflict of Interest

The authors declare that the research was conducted in the absence of any commercial or financial relationships that could be construed as a potential conflict of interest.

## Publisher’s Note

All claims expressed in this article are solely those of the authors and do not necessarily represent those of their affiliated organizations, or those of the publisher, the editors and the reviewers. Any product that may be evaluated in this article, or claim that may be made by its manufacturer, is not guaranteed or endorsed by the publisher.

## Abbreviations

ALT, Alanine Aminotransferase; ALB, Albumin; ALP, Alkaline Phosphatase; AMY, Amylase; BUN, Blood Urea Nitrogen; BSA, Bovine Serum Albumin; Ca^2+^, Calcium Ion; CCA, Canonical Correspondence analysis; Cl^-^, Chloride; CHOL, Cholesterol; GLO, Globulin; GLU, Glucose; HS, Heat Stress; HE, Hematoxylin-Eosin Staining; IBD, Inflammatory bowel disease; IL, Interleukin; IEC, Intestinal Epithelium Cells; KEGG, Kyoto Encyclopedia of Genes and Genomes; LAC, Lactic Acid; LEfSe, line discriminant analysis effect size; NF-κB, Nuclear Factor κ Appa B; OTU, Operational taxonomic unit; PAS, Periodic Acid-Schiff Stain; PBS, Phosphate buffer saline; K+, Potassium Ion; PCA, Principal Component Analysis ; PCoA, Principal Co-ordinates Analysis; RDA, Redundancy analysis; sIgA, Secretory Immunoglobulin A; SCFAs, Short Chain Fatty Acids; Na^+^, Sodium Ion; SPF, Specific Pathogen Free; TBIL, Total Bilirubin; tCO2, Total Carbon Dioxide; TP, Total Protein; WGCNA, Weighted Gene Co-Expression Network Analysis.
