# Supplementary material for: A Comprehensive Analysis of the Colonic Flora Diversity, Short Chain Fatty Acid Metabolism, Transcripts, and Biochemical Indexes in Heat-Stressed Pigs
Source: Front Immunol. 2021 Oct 21;12:717723. doi: 10.3389/fimmu.2021.717723 (PMC8567839; doi:10.3389/fimmu.2021.717723)
Supplement: Supplementary file 1 [file DataSheet_1.pdf]

Main reagents

Phosphoric acid (Chinese National Medicines), diethyl ether (Chinese National Medicines), acetic acid (sigma⩾99.5%), propionic acid (sigma>99.0%), butyric acid (sigma>99.0%), isobutyric acid (sigma>99.0%), valeric acid (sigma) >98.0%), isovaleric acid (sigma>99.0%), caproic acid (Aladdin⩾99.5%), isohexanoic acid (sigma>98%).

Standard sample preparation

Measure appropriate amounts of pure standards of acetic acid, propionic acid, butyric acid, isobutyric acid, valeric acid, isovaleric acid, and caproic acid, and prepare them with ether to make 0.02 μg / mL, 0.05 μg / mL, 0.1 μg / mL, 0.2 μg /mL, 0.5 μg / mL, 1 μg / mL, 2 μg / mL, 5 μg / mL, 10 μg / mL, 25 μg / mL, 50 μg / mL, 100 μg / mL, 250 μg / mL, 500 μg / mL mixed standards of concentration gradient. The working standard solutions are stored at 0 ℃.

Sample pretreatment

50 μl of 15% phosphoric acid were added to 50 mg of colonic content, add more 100 μL 125μg/ml internal standard (isohexanoic acid) solution, and 400 μL ether, tissue homogenate for 1 min, then centrifugation at 4 ° C and 12000 × g for 10 min.

GC-MS detection

The supernatant was analyzed by gas chromatography-mass spectrometry (GC-MS, Agilent Technologies Inc., Agilent 6890N/5975B, USA). The column used was Agilent HP innowax capillary column (30 m × 0.25 mm ID × 0.25 μm) in the split injection mode. The injection volume was 1 μL and split ratio was 10:1. The temperatures of the injection port, ion source, line, and quadrupole were 250 ℃, 230 ℃, 250 ℃, and 150 ℃, respectively. The initial temperature of the programming was 90 ℃. The temperature was raised to 120 ℃ (at 10 ℃/min), followed by an increase to 150 ℃ (at 5 ℃/min). Finally, the temperature was raised to 250 ℃ for 2 min (at 25 ℃/min). The carrier gas was helium with a flow rate of 1.0 mL/min. The mass spectrometry used an electron impact ionization source and SIM scanning mode, and

the electron energy was 70 ev. Based on the detection results, target quantification of the detected samples

Total Ion Chromatogram

From the performance of the TIC (Total Ion Chromatogram) chart, all 8 short-chain fatty acids can be distinguished. The peak time of the internal standard (isohexanoic acid) is 9.37 minutes of standard samples, which is clearly separated from other short-chain fatty acid standards, indicating that the method is good (Fig S1).

After the concentration series of the standard solution is measured, the concentration of the standards are taken as the abscissa, and the ratio of the peak area of the standard for the internal standard is taken as the ordinate to construct a calibration curve. The linear regression equation of each substance is shown in the following table S1 (correlation coefficient $R > 0.99$). A standard sample with a mixed standard concentration of 25 μg / mL was injected eight times in a row, and the intra-day precision was calculated, expressed as relative standard deviation (RSD).

A standard sample of 25 μg/mL is processed every day for determination on the first, second, and third day, and the inter-day precision is calculated, expressed as relative standard deviation (RSD). The intra-day precision is between 0.71% and 2.83%, and the intra-day precision is between 3.46% and 12.41%, indicating that the precision of the instrument is good (Table S1).

Repeat six samples to obtain the repeatability of concentration calculation, expressed shown in RSD (Table S1). The recovery results show that each substance has a good linearity within the concentration range. The intra-day and inter-day precision and repeatability are less than 15%, and the recovery rate is between 80%-115%, which meets the analysis requirements of the sample, indicating that this method is stable and reliable. It can be applied to the detection of samples (Table S2). After quality control analysis, perform concentration quantification (Fig. S1C).

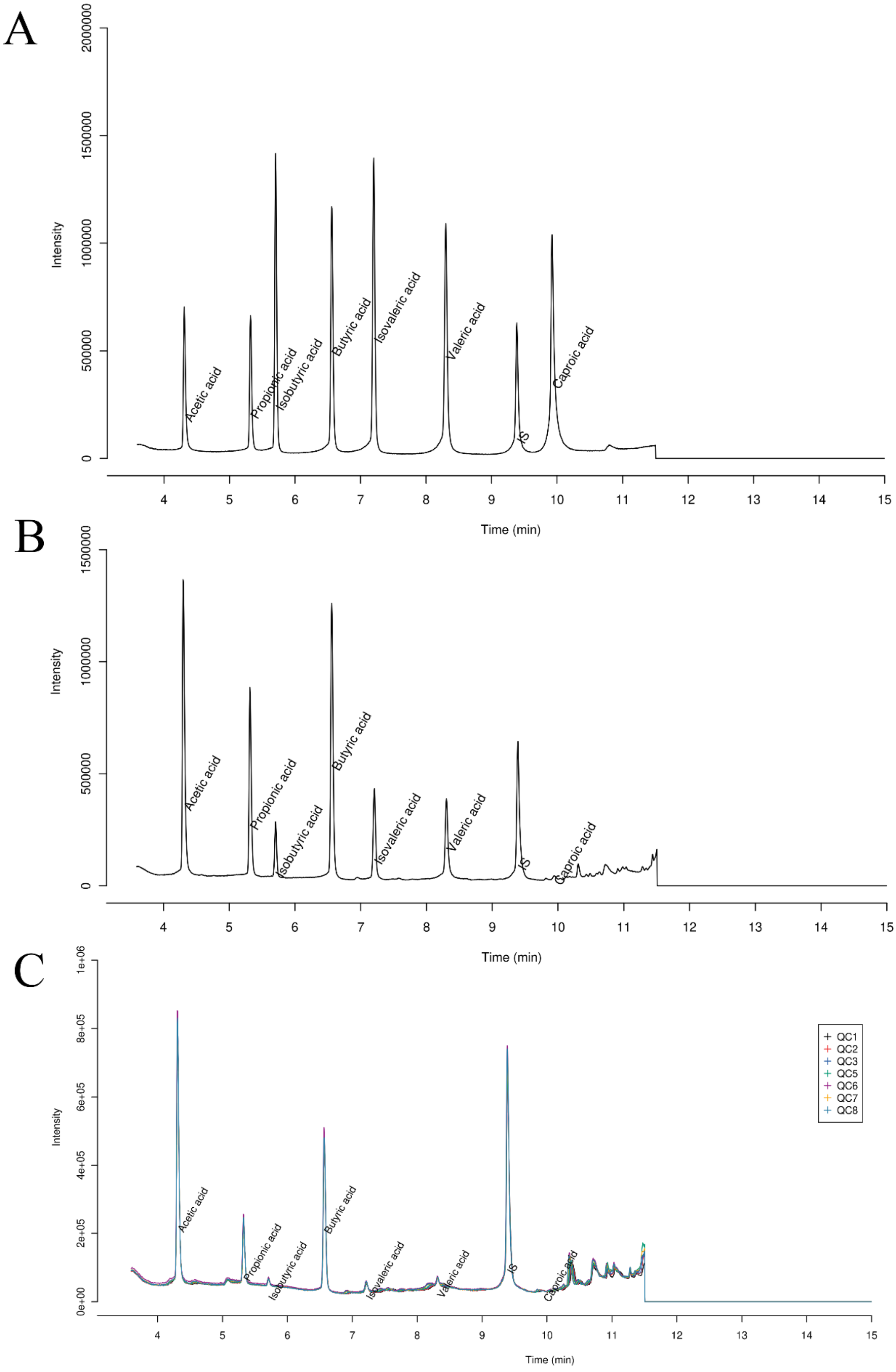

Fig S1. Total ion chromatogram

A. Mixed standard TIC graph; B. Sample TIC graph, C. Mixed QC sample overlapping chromatogram

Table S1. Linear regression equation, precision, repeatability and limit of quantification of standard samples

SCFAs	CAS	Quantitative ion	Linear equation	Correlation coefficient（R）	Linear range(μg/mL)	Intraday precision RSD (%)	within-day precision RSD (%)	Repeatability RSD (%)
Acetic acid	64-19-7	60	y=0.0181x + 0.0342	0.9956	0.02~500	1.85	11.73	5.44
Propionic acid	79-09-4	74	y=0.0208x + 0.0035	0.991	0.02~500	1.73	5.28	4.72
Isobutyric acid	79-31-2	73	y=0.0233x + 0.0016	0.9935	0.02~500	1.31	4.76	5.65
Butyric acid	107-92-6	60	y=0.0571x + 0.0149	0.9967	0.02~500	1.57	6.87	5.51
Isovaleric acid	503-74-2	60	y=0.0656x + 0.0146	0.9955	0.02~500	1.35	6.63	7.59
Valeric acid	109-52-4	60	y=0.064x + 0.0126	0.9969	0.02~500	1.3	5.66	7.68
Caproic acid	142-62-1	60	y=0.0573x - 0.0032	0.9973	0.02~500	1	7.71	9.53

Table S2. Standard sample recovery results

	Acetic acid	Propionic acid	Isobutyric acid	Butyric acid	Isovaleric acid	Valeric acid	Caproic acid
LQC (μg / mL)	1	1	1	1	1	1	1
Recovery rate	90.34%	94.55%	107.07%	96.35%	92.82%	99.25%	85.22%
MQC (μg / mL)	25	25	25	25	25	25	25
Recovery rate	97.25%	87.81%	86.17%	85.56%	85.50%	88.16%	96.97%
HQC (μg / mL)	100	100	100	100	100	100	100
Recovery rate	106.31%	87.53%	85.20%	86.38%	85.80%	89.19%	96.76%
