# Supplementary material for: A Comprehensive Analysis of the Colonic Flora Diversity, Short Chain Fatty Acid Metabolism, Transcripts, and Biochemical Indexes in Heat-Stressed Pigs
Source: Front Immunol. 2021 Oct 21;12:717723. doi: 10.3389/fimmu.2021.717723 (PMC8567839; doi:10.3389/fimmu.2021.717723)
Supplement: Supplementary file 2 [file DataSheet_2.pdf]

Name	sample No.	conc. (ng/ul)	volu. (ul)	weight (ug)	QC result *	Margin of Sample	Unavailable Cause
C-01-1	BMK210726-X925-020001-01	2396.1	45.0	107.8	A	Sufficient	
C-01-2	BMK210726-X925-020002-01	1658.9	45.0	74.7	A	Sufficient	
C-01-3	BMK210726-X925-020003-01	2223.9	45.0	100.1	A	Sufficient	
C-01-4	BMK210726-X925-020004-01	2052.7	45.0	92.4	C	Sufficient	Integrity
C-07-1	BMK210726-X925-020005-01	1472.5	35.0	51.5	C	Sufficient	Integrity
C-07-2	BMK210726-X925-020006-01	1898.1	45.0	85.4	C	No surplus	Integrity
C-07-3	BMK210726-X925-020007-01	1041.6	27.0	28.1	C	No surplus	Integrity
C-07-4	BMK210726-X925-020008-01	2194.7	45.0	98.8	A	Sufficient	
C-14-1	BMK210726-X925-020009-01	2480.9	55.0	136.4	C	Sufficient	Integrity
C-14-2	BMK210726-X925-020010-01	1880.3	22.0	41.4	C	Sufficient	Integrity
C-14-3	BMK210726-X925-020011-01	1361.5	45.0	61.3	A	Sufficient	
C-14-4	BMK210726-X925-020012-01	2222.0	45.0	100.0	C	Insufficient	Integrity
C-21-1	BMK210726-X925-020013-01	1607.0	22.0	35.4	C	Insufficient	Integrity
C-21-2	BMK210726-X925-020014-01	2806.5	45.0	126.3	A	Sufficient	
C-21-3	BMK210726-X925-020015-01	1710.4	45.0	77.0	A	Insufficient	
C-21-4	BMK210726-X925-020016-01	1422.3	45.0	64.0	A	Insufficient	

Note*:

The test conclusion is based on the comprehensive evaluation of the tested samples based on the requirements of the Beijing Biomarker sequencing sample test standard;

A: Qualified, the quality meets the requirements of building a database, and the total amount meets 3 times or more of the conventional amount of building a database;

B+: The quality meets the requirements of building a database, and the total amount meets 2 regular volume building of the database

B-: The quality meets the requirements for building a database, and the concentration and total amount are less than the requirements for building a database for 2 times

C: The sample quality does not fully meet the requirements for library construction and sequencing, and the samples that can be built at risk but not guaranteed to meet the contract requirements

D: The sample quality does not completely meet the requirements for library construction and sequencing, and it is not recommended to use samples

Sample inspection quality table

Sample Name	No.	RIN vaule	28S/18S	Baseline	OD260/280	OD260/230	Sample status	Dilution times
C-01-1	1	8.5	3.2	normal	2.07	1.8	normal	24
C-01-2	2	8	2.6	normal	2.05	2.05	normal	17
C-01-3	3	7.8	2.32	normal	2.04	2.08	normal	22
C-01-4	4	7.7	1.98	unavailable	2.04	1.9	normal	21
C-07-1	5	7.4	1.68	unavailable	2.07	2.13	normal	15
C-07-2	6	7	1.43	unavailable	2.03	2.1	normal	19
C-07-3	7	6.4	0.92	unavailable	2.01	2.19	normal	10
C-07-4	8	7.8	2.23	Partially available	2.05	1.98	normal	22
C-14-1	9	7.6	2.45	unavailable	2.06	1.89	normal	25
C-14-2	10	7.1	1.79	unavailable	2.05	2.19	normal	19
C-14-3	11	8.3	2.89	normal	2.01	2.03	normal	14
C-14-4	12	7.3	1.87	unavailable	2.03	2.08	normal	22
C-21-1	13	7.3	1.88	unavailable	2.03	2.17	normal	16
C-21-2	14	8.2	2.82	normal	2.06	1.98	normal	28
C-21-3	15	8	2.62	normal	2.03	2.09	normal	17
C-21-4	16	7.7	2.35	normal	2.02	2.1	normal	14

2、Peak Graph of Nanodrop

Result of 2100

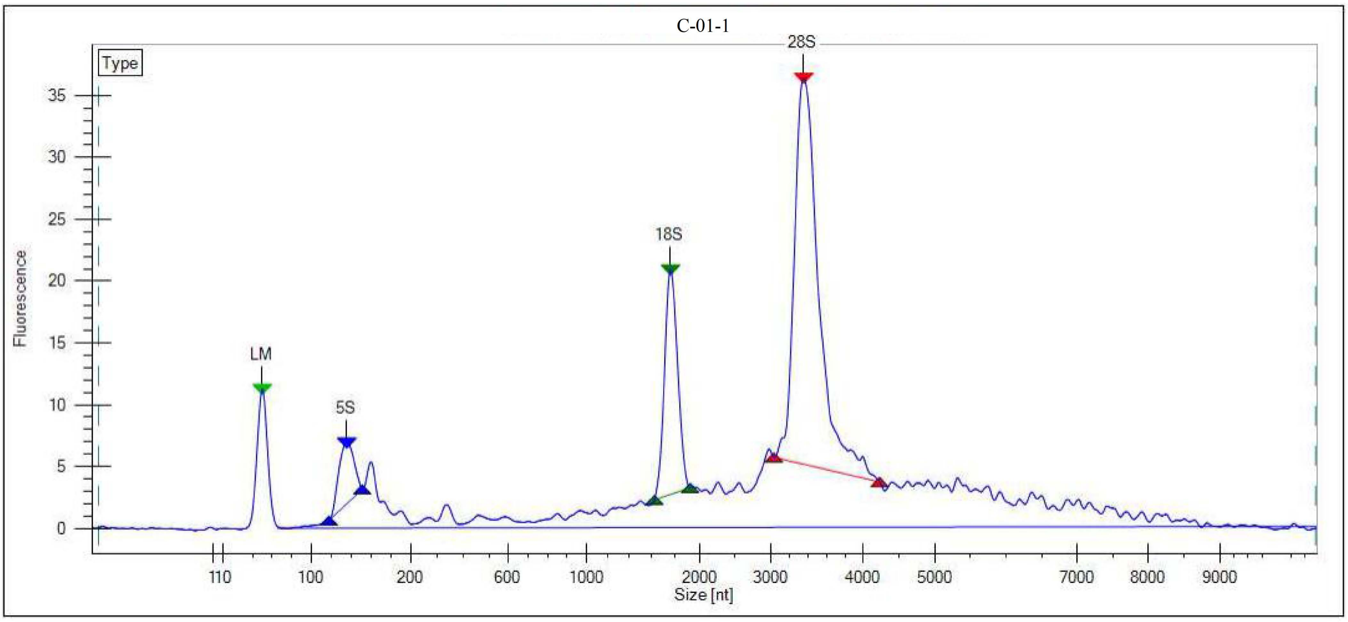

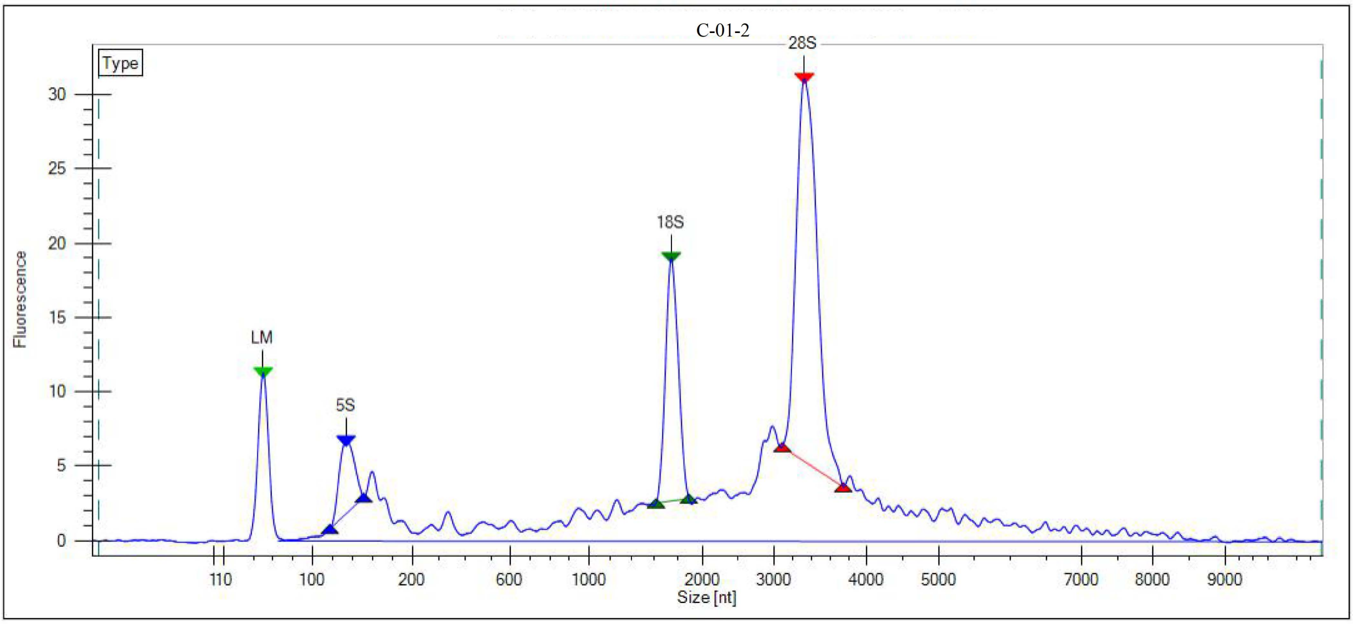

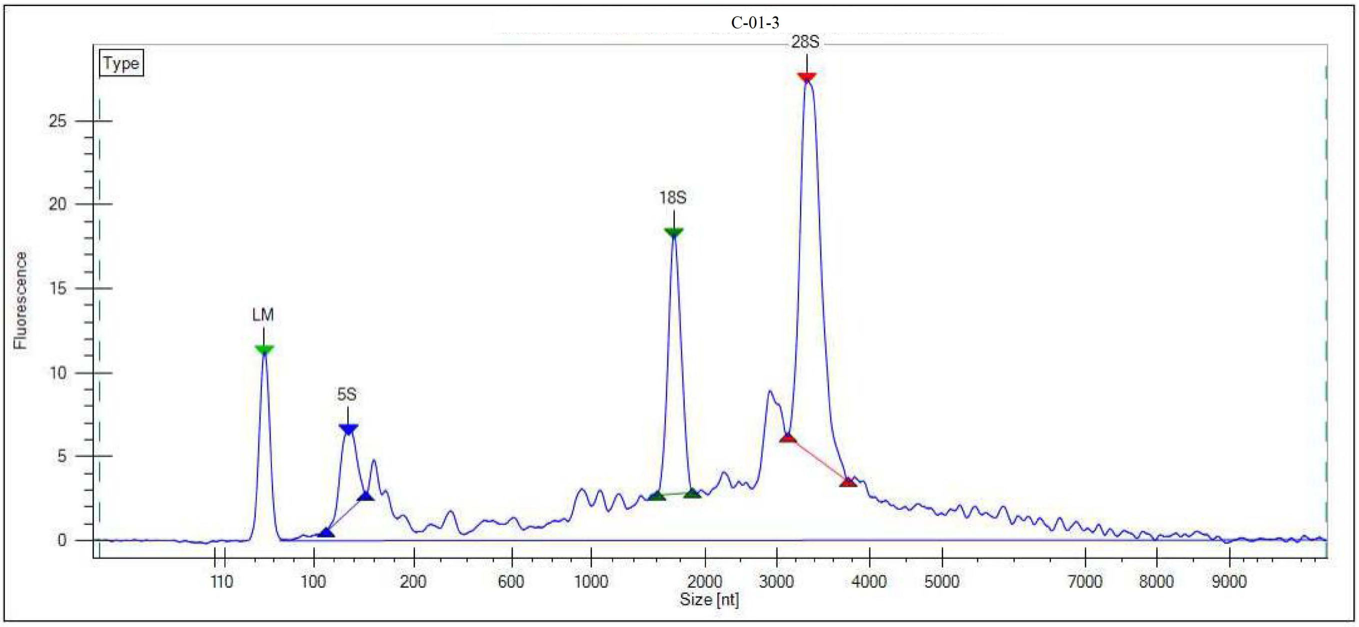

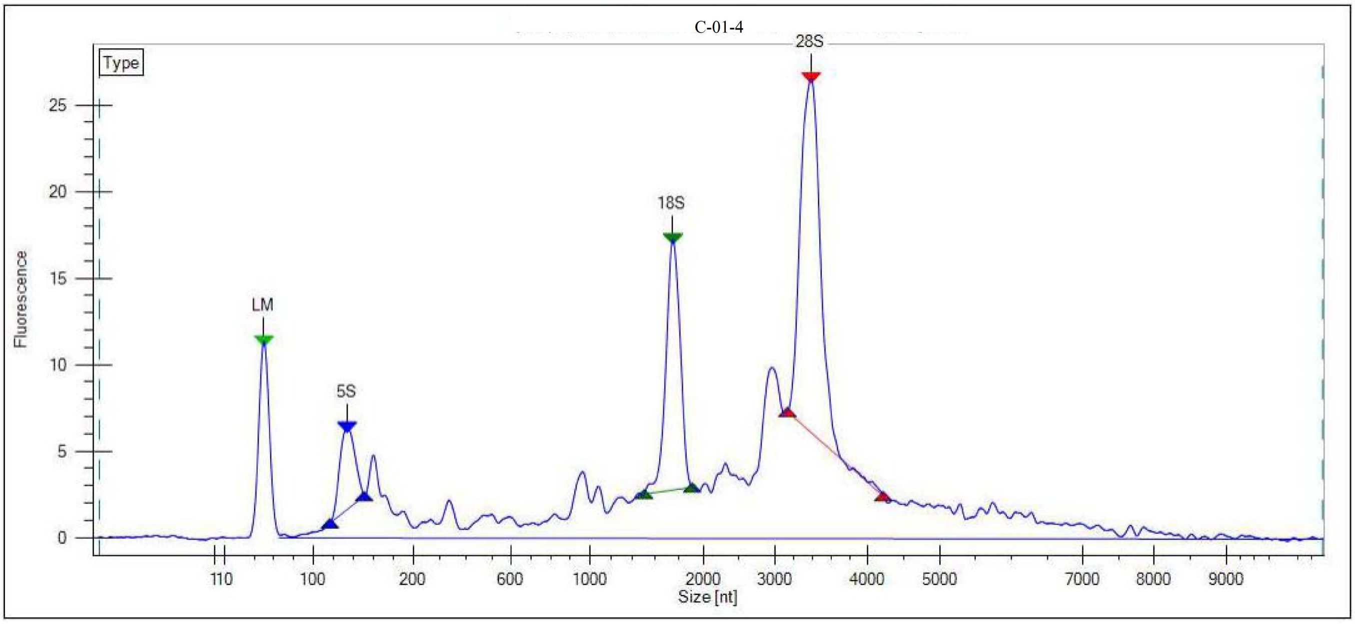
C-01-4
Type
28S
18S
LM
5S
Fluorescence
0
5
10
15
20
25
110
100
200
600
1000
2000
3000
4000
5000
7000
8000
9000
Size [nt]

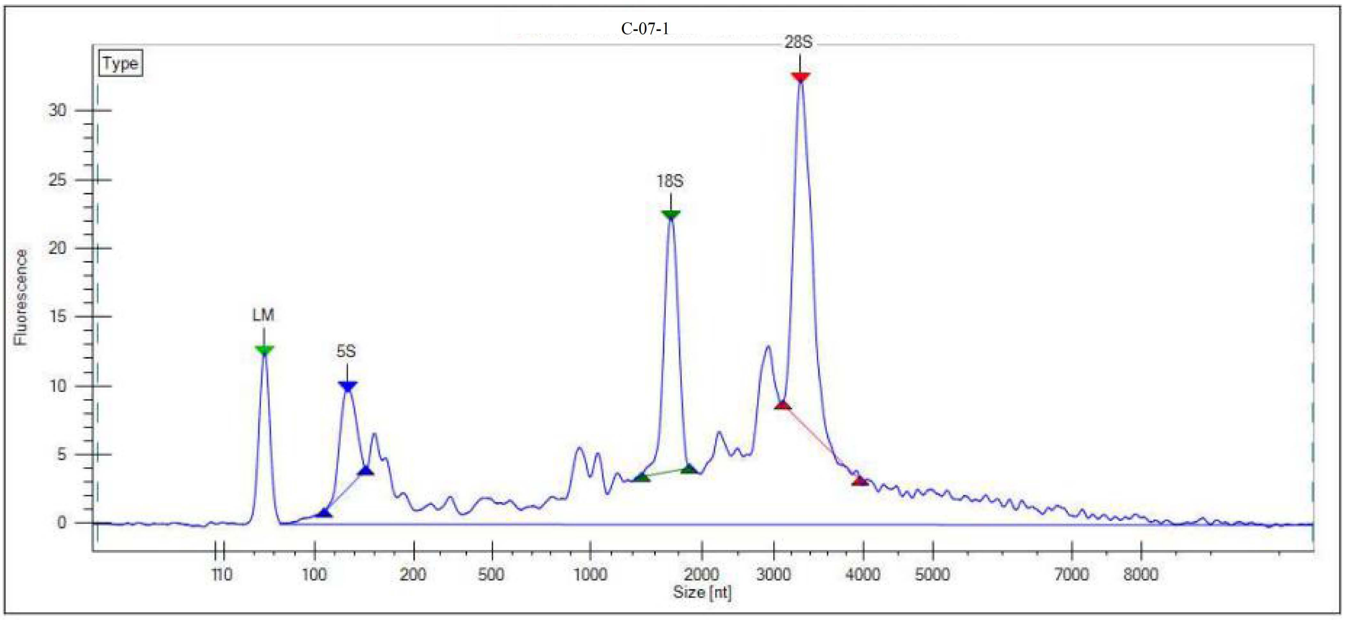
C-07-1
Type
28S
18S
LM
5S
Fluorescence
0
5
10
15
20
25
30
110
100
200
500
1000
2000
3000
4000
5000
7000
8000
Size [nt]

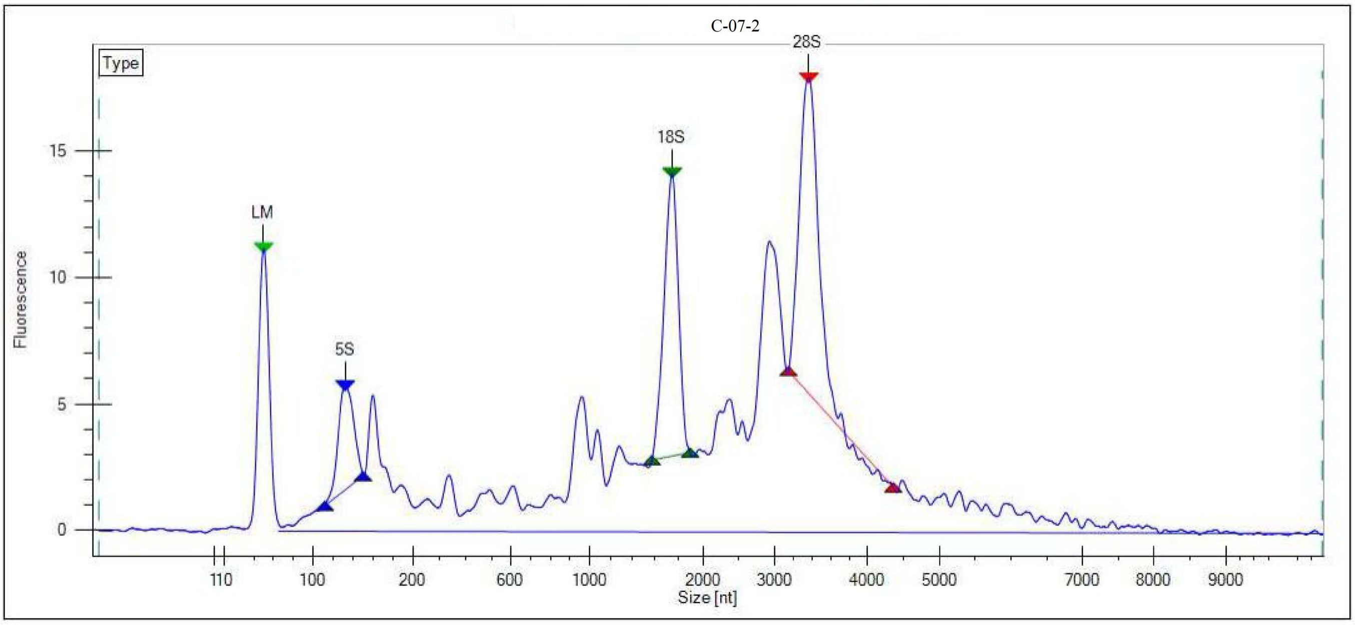
C-07-2
Type
28S
18S
LM
5S
Fluorescence
0
5
10
15
110
100
200
600
1000
2000
3000
4000
5000
7000
8000
9000
Size [nt]

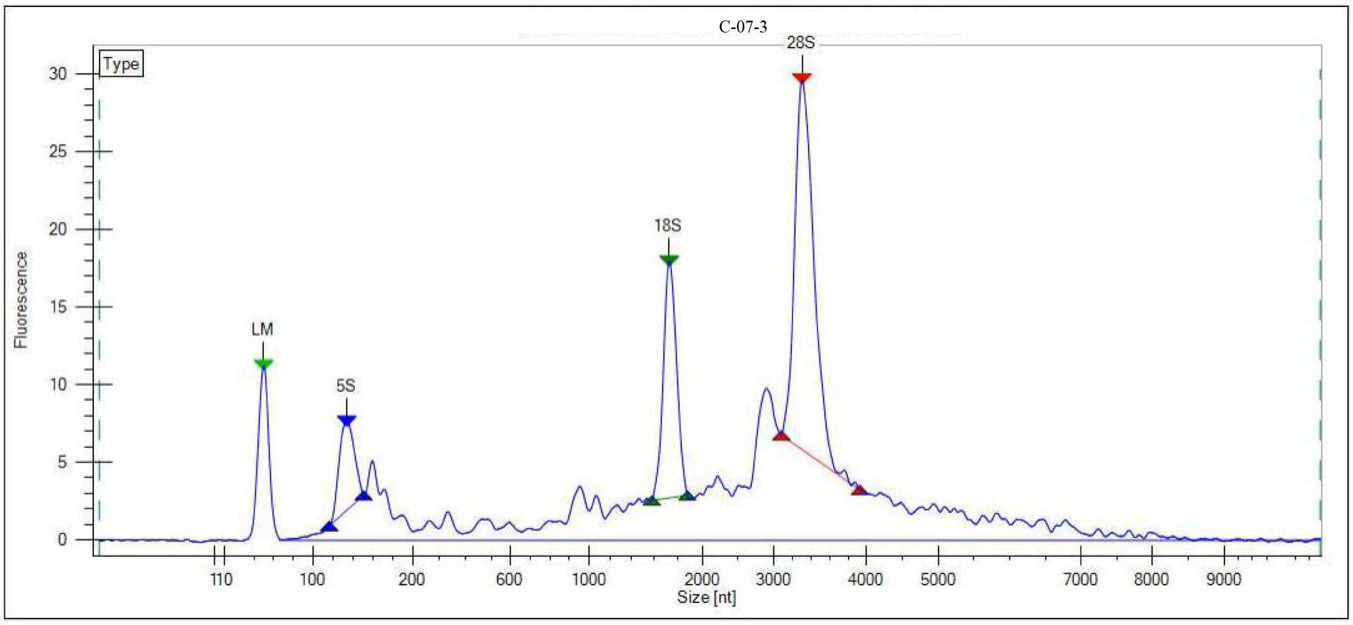

C-07-3
Type
28S
18S
LM
5S
Fluorescence
30
25
20
15
10
5
0
110
100
200
600
1000
2000
3000
4000
5000
7000
8000
9000
Size [nt]

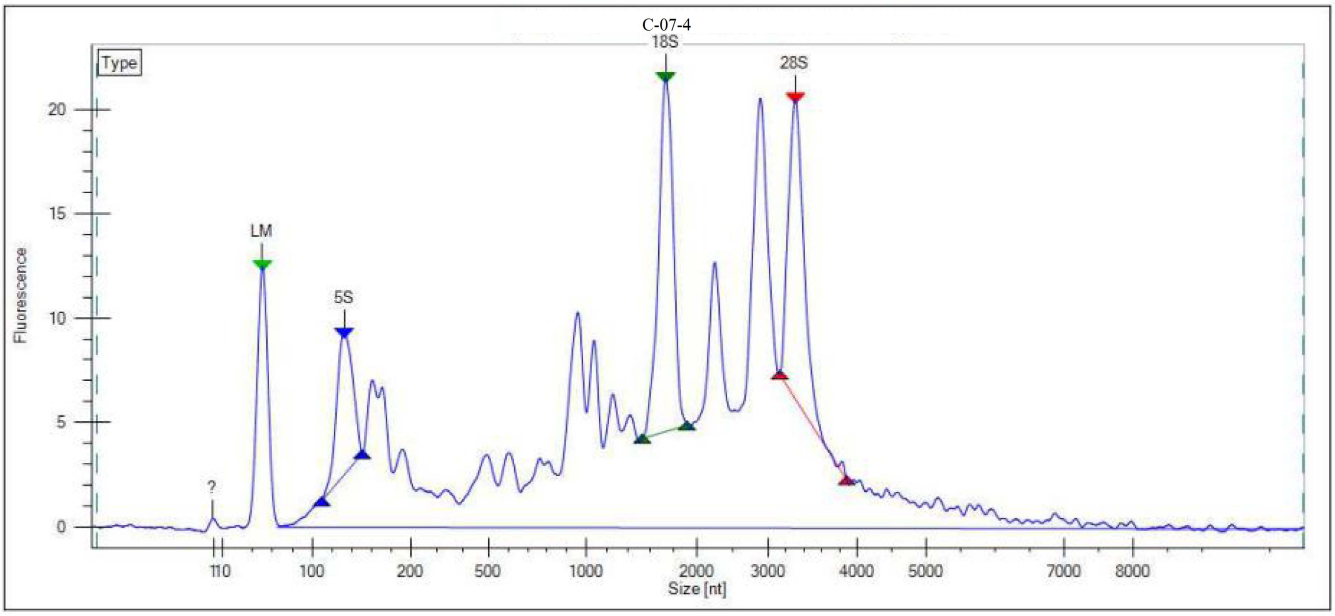

C-07-4
Type
18S
28S
LM
5S
?
Fluorescence
20
15
10
5
0
110
100
200
500
1000
2000
3000
4000
5000
7000
8000
Size [nt]

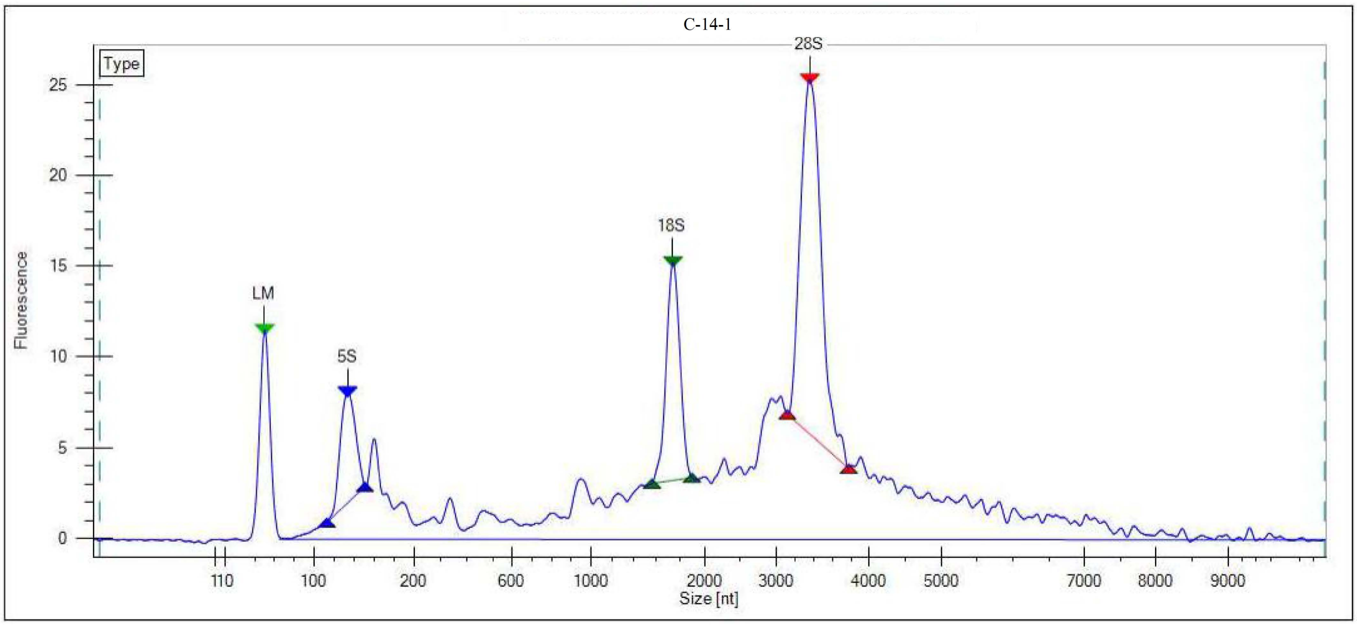

C-14-1
Type
28S
18S
LM
5S
Fluorescence
25
20
15
10
5
0
110
100
200
600
1000
2000
3000
4000
5000
7000
8000
9000
Size [nt]

C-14-2

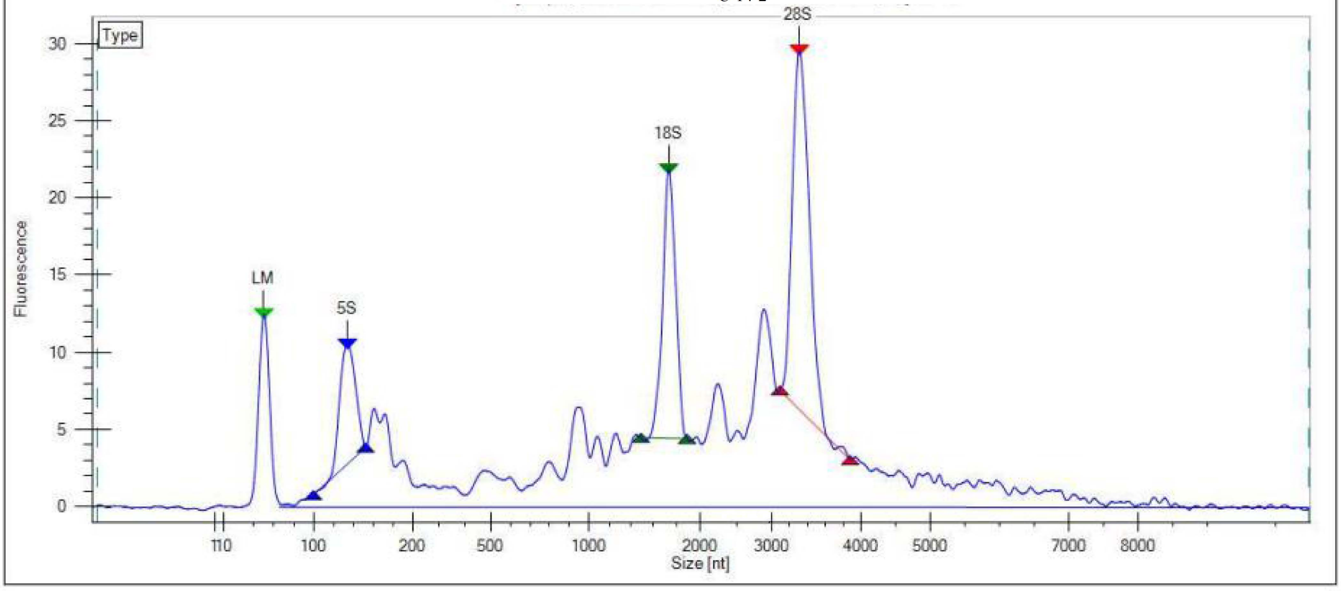

C-14-3

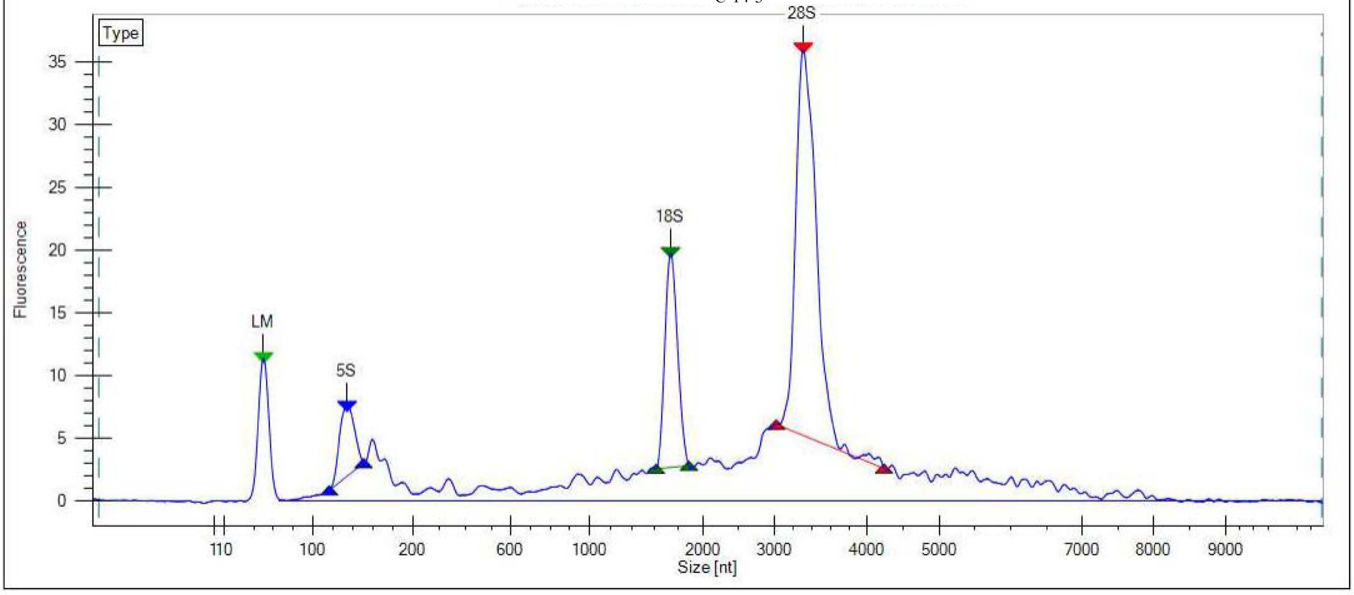

C-14-4

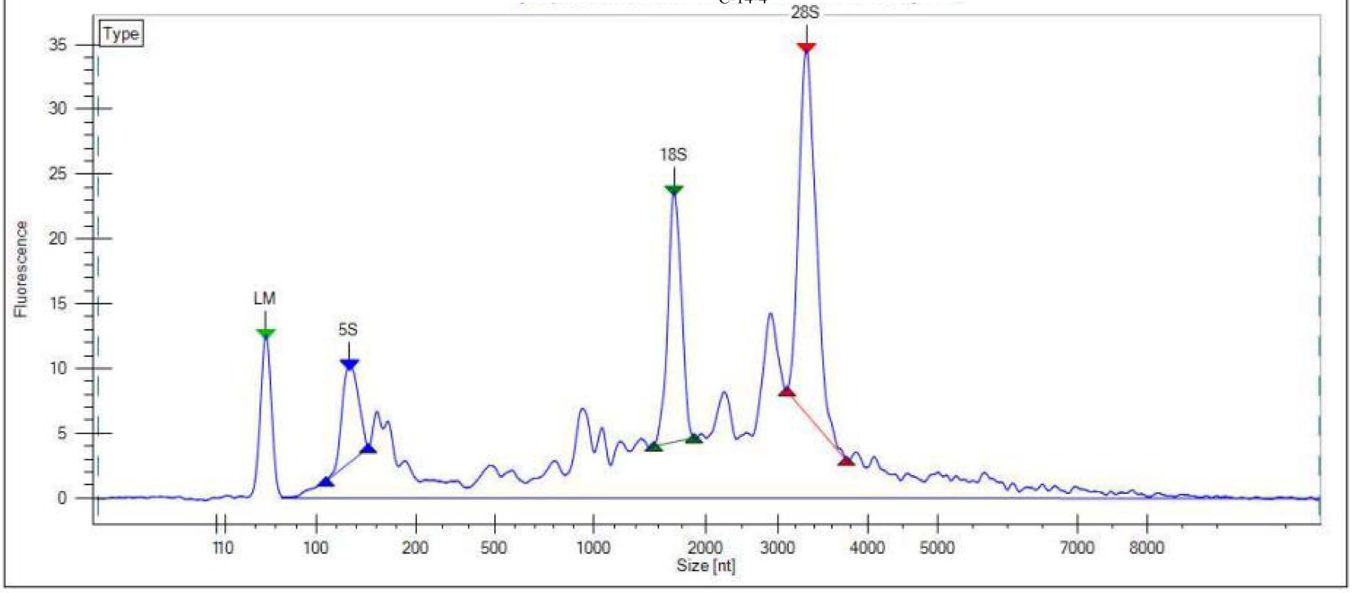

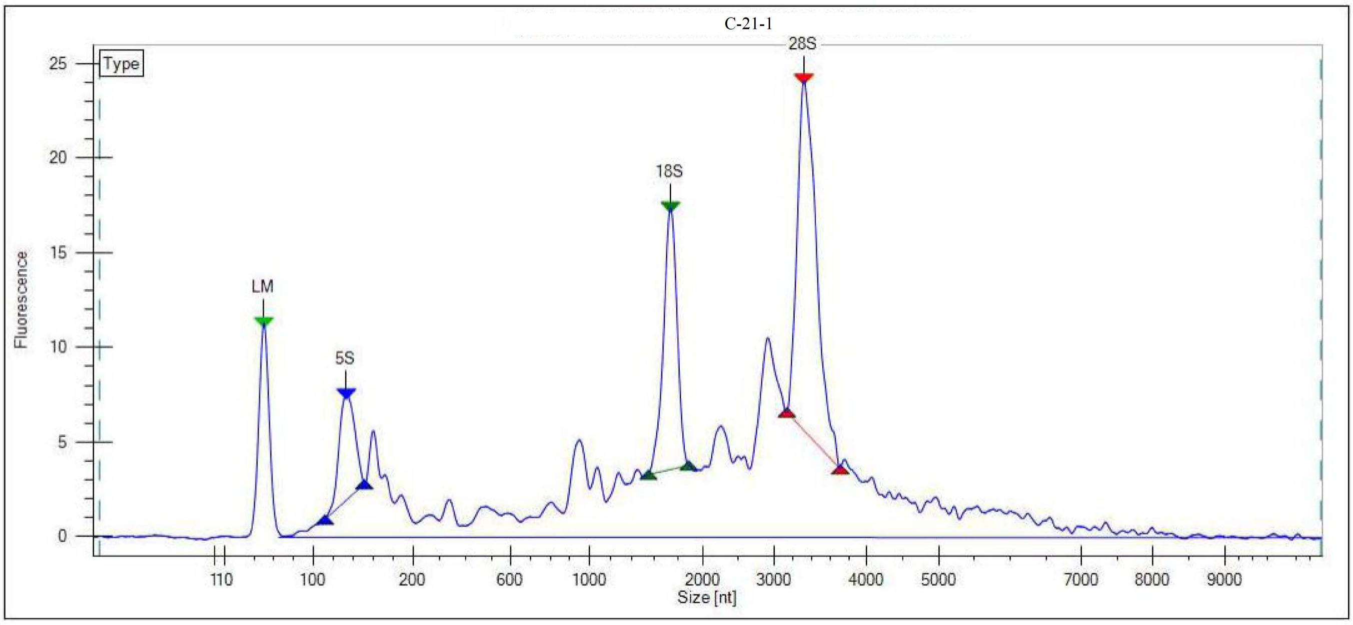
C-21-1
28S
18S
LM
5S
Type
Fluorescence
0
5
10
15
20
25
110
100
200
600
1000
2000
3000
4000
5000
7000
8000
9000
Size [nt]

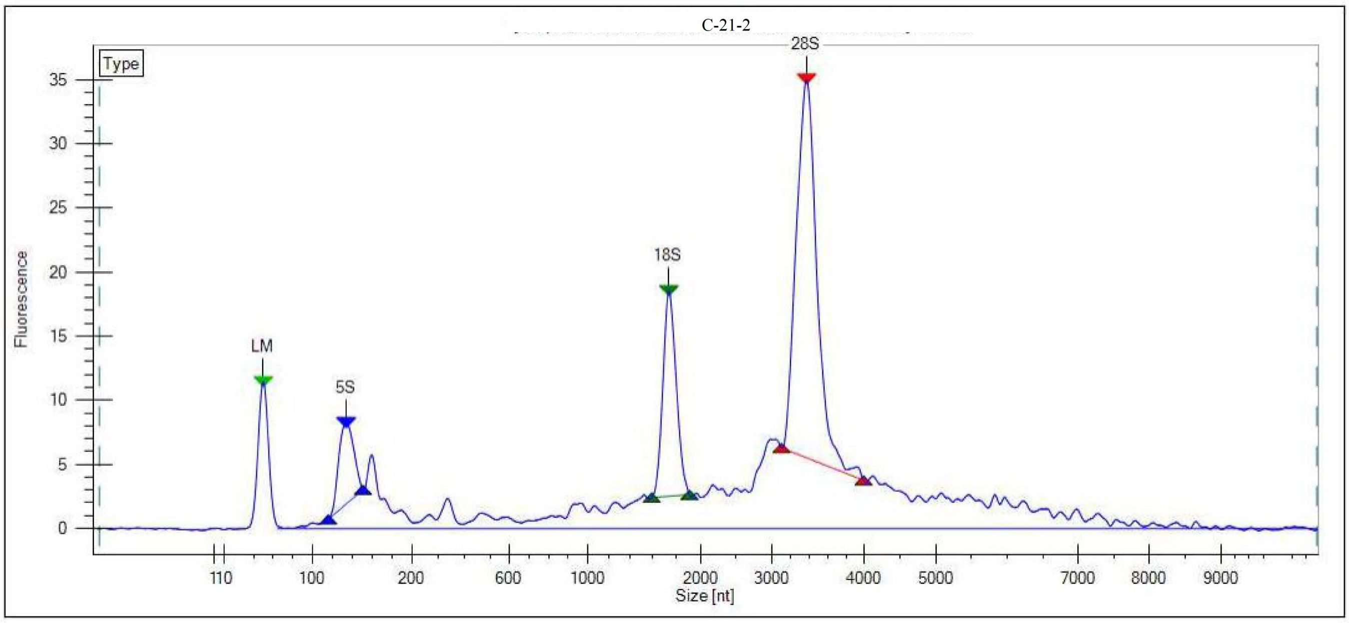
C-21-2
28S
18S
LM
5S
Type
Fluorescence
0
5
10
15
20
25
30
35
110
100
200
600
1000
2000
3000
4000
5000
7000
8000
9000
Size [nt]

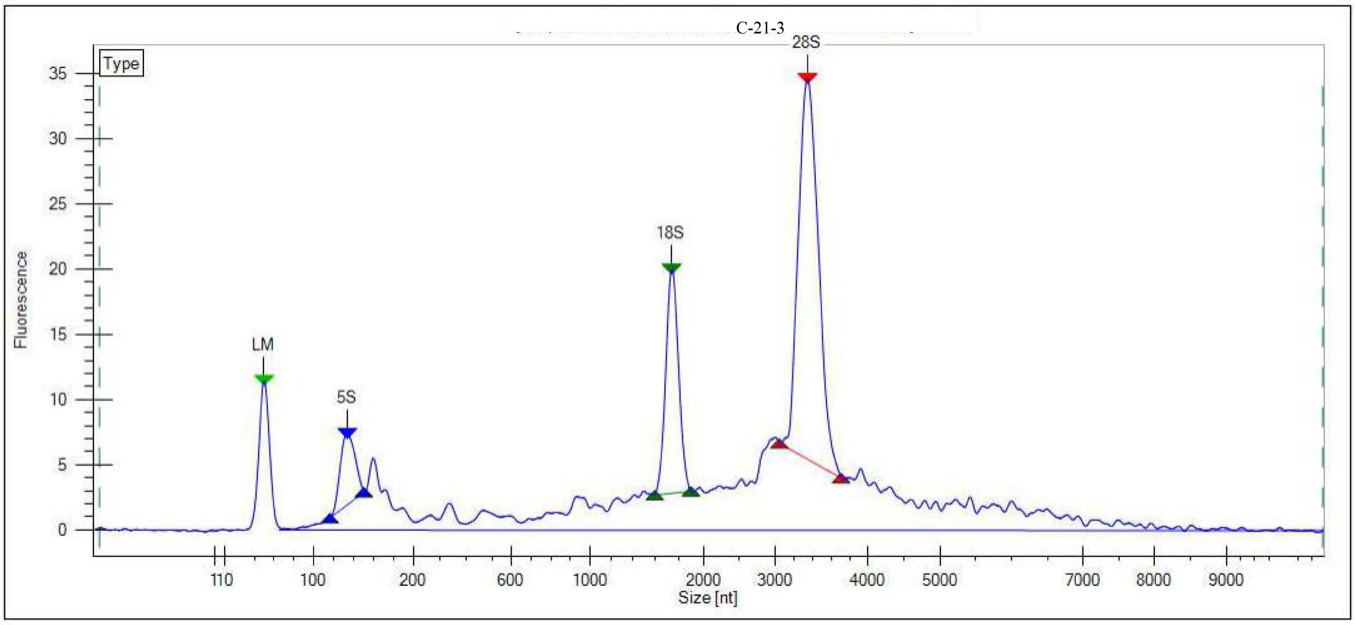
C-21-3
28S
18S
LM
5S
Type
Fluorescence
0
5
10
15
20
25
30
35
110
100
200
600
1000
2000
3000
4000
5000
7000
8000
9000
Size [nt]

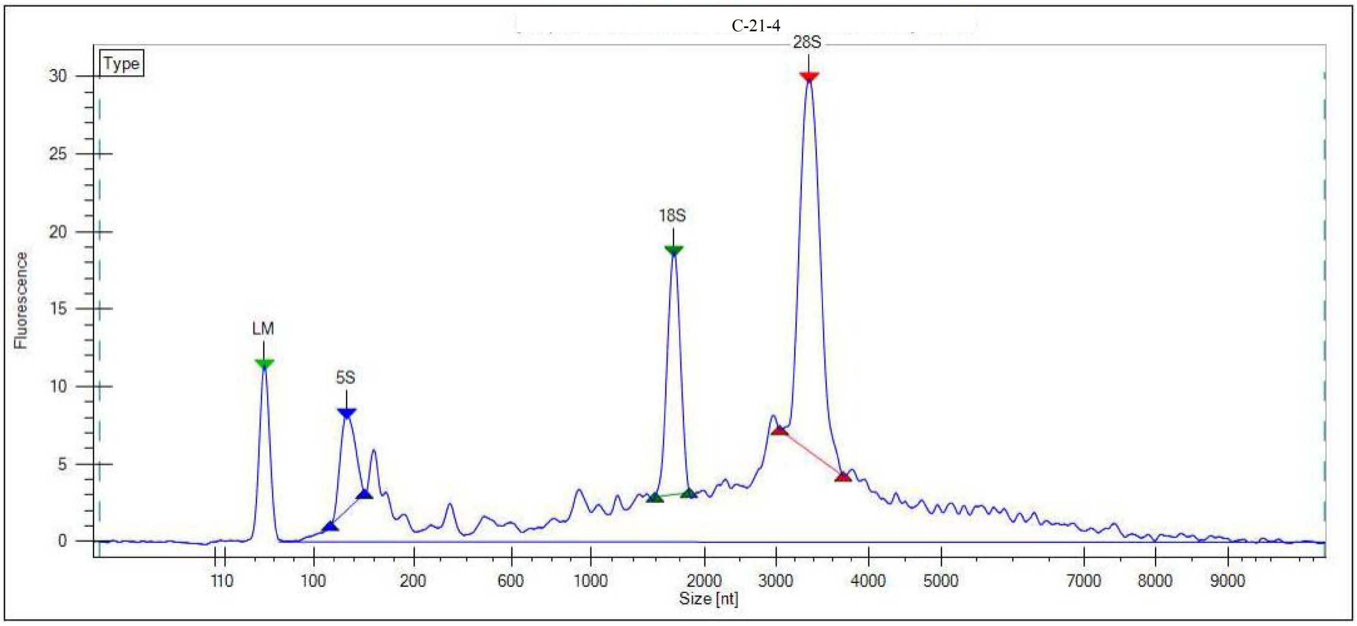
C-21-4
Type
28S
18S
LM
5S
Fluorescence
30
25
20
15
10
5
0
110
100
200
600
1000
2000
3000
4000
5000
7000
8000
9000
Size [nt]
